# Soil moisture precipitation feedbacks in the Eastern European Alpine region in convection‐permitting climate simulations

**DOI:** 10.1002/joc.8234

**Published:** 2023-09-10

**Authors:** Heimo Truhetz, Aditya N. Mishra

**Affiliations:** ^1^ Wegener Center for Climate and Global Change (WEGC) University of Graz Graz Austria

**Keywords:** CCLM, climate, convection, convection permitting, land‐atmosphere, pseudo‐global warming

## Abstract

A novel convection permitting modelling framework that combines a pseudo‐global warming approach with continuously forced deep soil moisture from prescribed perturbation storylines is applied in the Eastern European Alpine region and parts of the Pannonian Basin to investigate soil moisture precipitation (SMP) feedbacks on summertime precipitation and the feedbacks’ role under changed climate conditions. A set of 1‐year convection‐permitting (3 km horizontal grid spacing) soil moisture sensitivity simulations with the regional climate model of the Consortium for Small‐Scale Modelling in Climate Mode are conducted. In order to account for global warming, end‐of‐the‐century climate change effects from four global climate models, projecting the greenhouse gas concentration scenario RCP 8.5, are imprinted. The simulations reveal that (1) the locations of precipitation events are highly sensitive to soil moisture modifications while intensities and the internal structure of precipitation events are nearly unaffected and (2) high precipitation intensities are more likely in combinations with positive temporal but distinctive (either strong positive or strong negative) spatial SMP coupling. Low precipitation intensities are in favour of combinations of negative temporal and positive spatial coupling. The analyses suggest that soil moisture at a given time acts as a guiding field for the location of the next precipitation event. Interestingly, this behaviour is independent of climate change, although the coupling strength's increase is 1.5–1.7 times larger than expected from linear climate change scaling when climate becomes 50% dryer. Finally, it is found that (1) local deviations in the climate change signal of summertime precipitation in the range of up to ±40% are caused by uncertainty in deep soil moisture in the range of ±10% and (2) these local deviations in the climate change signal are dominated by soil moisture uncertainty in future climate conditions.

## Graphical abstract

In a PGW framework (current, more humid, and dryer climates), heavy summertime precipitation events from the Eastern Alpine region to the Western Pannonian Basin prefer positive temporal with positive/negative spatial SMP feedbacks in a CPM. Uncertainty in local climate change is dominated by deep soil moisture uncertainty in future climates.

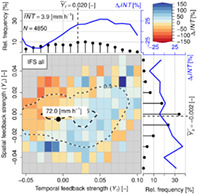

## 1 INTRODUCTION

**FIGURE 1:**
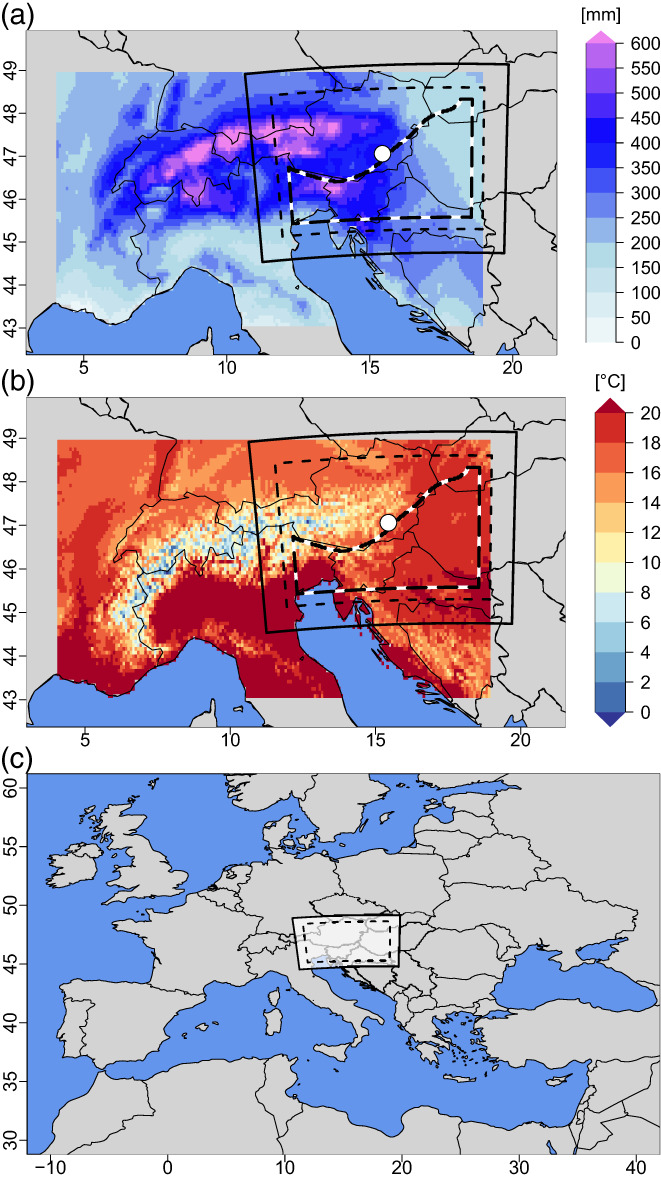
Geographical and climatological conditions of the study area. (a) mean summertime (JJA) precipitation sum [mm] and (b) mean JJA temperature [°C] of the period 1971–2000, derived from gridded station data HISTALP (Auer et al., 2007), together with the modelling domain (solid lined polygon), its lateral sponge zone of 20 grid cells (dashed polygon), and the study area (b/w dashed polygon) that starts at the City of Graz (white dot) and extends to the South and East; (c) position of the modelling domain (white rectangle) on a European map. [Colour figure can be viewed at wileyonlinelibrary.com]

Precipitation in the European Alpine region shows large variability ranging from 400 mm per year (in low elevated areas) to 3000 mm per year (in the area of the highest summits in the Western Alps; Isotta et al., 2014). In the Eastern Alpine region and its foothills, which turn into the Pannonian Basin further to the East, long‐term (period 1971– 2000) mean summertime precipitation and temperature change from about 350 mm and 18°C (Basin of Graz) to less than 200 mm and about 20°C to the East (Figure 1a, 1b), respectively. Since this region has seen significant temperature increases—multiple times larger than the global warming trend (Auer et al., 2007; Kabas et al., 2011)—and a gradual precipitation reduction over the last decades, it turned into a ‘hot spot’ (Koster et al., 2004) of land surface—atmosphere interaction. Indeed, based on artificial temperature and precipitation changes Hohmann et al. (2018) have demonstrated that future drought vulnerability (in the eastern foothills) will become more sensitive to climate change—even beyond current human adaptive measures.

In such regions, the soil moisture precipitation (SMP) feedbacks show complex behaviour. In a positive (more precipitation over wet soils) feedback loop, a wet soil leads to more moist static energy and convective available potential energy in the boundary layer (Eltahir, 1998) which favours deep convection in turn (Eltahir & Pal, 1996) and leads to a localised moisture recycling (Schar et al., 1999). For establishing a negative (more precipitation over dry soils) spatial feedback, spatial heterogeneity in soil moisture has been found to be the most important factor (Taylor et al., 2011). Deep convection is triggered at locations with strong soil moisture gradients and causes rainfall over dry areas (Taylor, de Jeu, et al., 2012). But spatial heterogeneity may also support the development of an enveloping larger‐scale positive temporal feedback (Guillod et al., 2015). Finally, since moisture transport from deeper soil layers to the surface takes time, the soil acts as a water storage that releases moisture with delay. This leads to a memory effect (Seneviratne et al., 2006) and introduces temporal feedbacks on various time scales, depending on the depths of soil layers (Wu & Dickinson, 2004). For instance, Yang et al. (2016) have shown that summer precipitation anomalies in Central Europe are caused by spring soil moisture patterns over Eastern Europe to a reasonable extent. Or Boé (2013) detected changing temporal SMP feedbacks in France in conjunction with distinctive large‐scale weather patterns and their lead time of occurrence of up to 15 days. But also the European summertime heatwave 2003 was supported by a lack of precipitation in winter and spring (Fischer et al., 2007; Teuling et al., 2013). A favour for positive temporal and negative spatial SMP feedbacks on larger scales (areas on the order of 250,000 km^2^) in Africa, South America, Australia, and other regions have been found by Guillod et al. (2015) on the basis of global remote sensing data. Yang et al. (2018) were additionally able to attribute the majority of negative feedbacks to arid and humid climate zones. Hsu et al. (2017) showed that the favour for negative spatial feedbacks is reduced (and may even turn into a favour for positive feedbacks) when the soil becomes wetter.

Since current climate models have difficulties in capturing the full range of SMP feedbacks, the impact of SMP feedbacks on a climate change signal remains unclear. In global climate models (GCMs) soil moisture feedbacks have been found to control atmospheric circulation in a way that the transport of moisture into drylands is enhanced and a future loss of water is reduced (Zhou et al., 2021). On the other hand, GCMs are in favour of positive SMP feedbacks (Moon et al., 2019) and their results are dominated by the implementation of the land‐atmosphere coupling which controls the climate change signal of summer precipitation in Central Europe (Vogel et al., 2018), in turn. Even if regional climate models (RCMs) may reduce deficiencies from GCMs (Sørland et al., 2018; Torma et al., 2015), their resulting climate change projections are largely pre‐dominated by their driving GCMs (Addor et al., 2016; Déqué et al., 2007; Wilby & Dessai, 2010). Moreover, such standard RCMs may also suffer from uncertain soil moisture feedbacks—also in Central and Eastern Europe (Jach et al., 2022; Knist et al., 2017).

Promising results are expected from climate models that are operated on such high spatial resolutions that parameterisation schemes for deep convection can be switched off (Weisman et al., 1997). These so‐called ‘convection permitting models’ (CPM) have demonstrated added value in simulating convective precipitation multiple times (Ban et al., 2021; Fosser et al., 2015; Meredith et al., 2021; Piazza et al., 2019; Prein et al., 2015; Schär et al., 2020) as well as their ability to alter (Hohenegger et al., 2009) and reproduce the observed effects of SMP feedbacks (Hohenegger & Stevens, 2018; Taylor et al., 2013). Based on artificially perturbed soil moisture fields, CPMs help to explore the complex SMP feedback mechanisms (Baur et al., 2018; Graf et al., 2021; Henneberg et al., 2018; Knist et al., 2020; Schlemmer et al., 2012) and their interaction with larger scale meteorological phenomena (Froidevaux et al., 2014) or the orography (Imamovic et al., 2017). However, since CPMs are computationally expensive, such soil moisture sensitivity experiments are just long enough to capture the phenomenon of investigation. And even if the length would be extended to include global warming as it is done in the Flagship Pilot Study on convective phenomena over Europe and the Mediterranean (FPSCONV; Coppola et al., 2020) of the Coordinated Downscaling Experiment (CORDEX; Giorgi et al., 2009) of the World Climate Research Programme, the impact of SMP feedbacks on climate change signals would still be difficult to be analysed: (1) initial soil moisture perturbations would fade along model integration (Khodayar et al., 2015) and weaken the effects of interest and (2) climate change effects in larger scale atmospheric dynamics (stemming from driving GCMs) would interfere with the implemented soil moisture perturbations. One way to overcome such deficiencies is to make use of the storyline (Lloyd & Shepherd, 2020; Shepherd et al., 2018; Sillmann et al., 2021) or pseudo‐global warming (PGW) (Brogli et al., 2022; Schär et al., 1996) approach. This approach is designed to investigate climate change effects on a process level. For instance, it has been successfully applied by Maraun et al. (2022) to investigate various drivers (including climate change) for a disastrous widespread landslide event in the Eastern Alpine region. But it may also be applied for long‐term periods, as it was done by Prein et al. (2017) and Rasmussen et al. (2020) who separated thermodynamic from dynamic climate change effects in decadal CPM simulations covering large parts of North America.

Another difficulty with climate models (including CPMs) stems from the uncertainty in deep soil moisture. Although so‐called Regional Earth System models with sophisticated 3D subsurface hydrodynamics have been developed over the last decade (e.g., the Terrestrial System Modelling Platform; Shrestha et al., 2014), many RCMs and CPMs still employ free drainage conditions (i.e. overrunning soil water is removed) or simple groundwater recharge conditions (Barlage et al., 2015). However, in presence of SMP feedbacks, such simplifications might significantly underestimate the range of uncertainty in simulated climate change effects—on short, but also on seasonal time scales due to the soil's memory effect.

The objectives of this study are twofold: (1) to investigate SMP feedbacks in the eastern Alpine foothills and the Western Pannonian Basin under current and various (diverting) future climate conditions and (2) to give a first impression on the impact of deep soil moisture uncertainty on climate change effects in summertime precipitation. Both targets are being pursued by means of a novel modelling framework: 1‐year convection‐permitting sensitivity experiments with deep soil moisture forcing from prescribed perturbation storylines are conducted in a PGW setting based on Maraun et al. (2022). This makes use of the advantages of CPMs and avoids disturbing effects from large‐scale circulation changes. In order to circumvent a fading of soil moisture perturbations (that occurs if just the initial soil moisture is perturbed), the soil moisture from deep (below 4 m) layers is continuously forced throughout the simulation period—equivalent to a prescribed deep soil moisture storyline for systematic perturbations. This gives fully established SMP feedbacks and a controlled drying/wetting of upper (hydrologic active) soil layers from below. Section 2 provides a detailed description of the experimental setup. The interplay between temporal and spatial SMP feedbacks and summertime precipitation events for current and under future climate changes are discussed in Section 3 and 4, respectively. Impacts of soil moisture uncertainty on the climate change signal of summertime precipitation is analysed in Section 5. Conclusions are drawn in Section 6.

## 2 EXPERIMENTAL SETUP

In this study, the operational forecast model of the Consortium for Small‐Scale Modelling in Climate Mode (CCLM; Rockel et al., 2008) is operated across the Eastern Alpine region and the Western Pannonian Basin (Figure 1c) with a spatial resolution of 0.0275° (~3 km) in convection‐permitting mode, that is, without deep convection parameterisation. The number of grid cells, the vertical resolution, the parameterisation schemes used, and other essential information about the model configuration are given in Table 1. Note, apart from the domain size, this configuration has also been used in CORDEX‐FPSCONV, see Ban et al. (2021) and Pichelli et al. (2021) for first results. Lateral boundary conditions and initial conditions are derived from the Integrated Forecast System (IFS; Bechtold et al., 2008) of the European Centre for Medium‐Range Weather Forecasts that has a horizontal grid spacing of 0.225° (~25 km) and consists of 91 vertical levels. In order to achieve a temporal resolution of 3 h, the IFS analysis fields from the data assimilation system (at 0000, 0600, 1200, 1800 UTC) are combined with the alternate steps (at 0300, 0900, 1500, 2100 UTC) of the IFS forecast product (Piazza et al., 2019; Suklitsch et al., 2011).

**TABLE 1:** Model configuration and parameterisations of CCLM (version 5.0 clm 9).

Domain	Eastern Alpine region and Western Pannonian Plane
Horizontal grid spacing	0.0275° (~3 km)
Time step [seconds]	25
No. grid cells	220 × 160
No. vertical atmospheric levels	60 up to 22 km altitude (time invariant pressure based sigma coordinate system; Gal‐Chen & Somerville, 1975)
Output interval [hour]	1
Lateral sponge zone	20 grid cells
Radiation scheme	Ritter and Geleyn (1992)
Convection scheme	Tiedtke (1989) for shallow convection, only
Microphysics scheme	One‐moment scheme including graupel (Doms & Baldauf, 2013)
Planetary boundary layer scheme	Prognostic turbulent kinetic energy scheme with 2.5 order closure (Mellor & Yamada, 1982)
Land surface scheme	TERRA_ML (Rockel et al., 2008) with 10 soil layers
Lower boundary of soil layers [m b. g. l.]	0.01, 0.04, 0.10, 0.22, 0.46, 0.94, 1.90, 3.82, 7.66, 15.34
Land use data	GLC2000 (Bartholomé & Belward, 2005)
Soil data	Digital Soil Map of the World (FAO‐Unesco, 1981)

In order to mimic various possible future changes, climate change signals from four GCMs (HadGEM2‐CC, IPSL‐CM5A‐MR, MIROC‐ESM, and GFDL‐ESM2M) of the CMIP5 (Taylor, Stouffer, & Meehl, 2012) ensemble, employing the Representative Concentration Pathway RCP 8.5, are derived from the periods 2071–2100 and 1985–2005 and imprinted onto the initial and lateral boundary conditions given by IFS: for each GCM and for each day of the year, a vertical column (averaged across the CCLM modelling domain for each pressure level) of mean climate change signals for temperature, relative humidity (changes in specific humidity are calculated from modified temperature, relative humidity, and pressure), and pressure is derived from the GCM data and added to IFS in a hydrostatic balanced way (Kröner et al., 2017). Additionally, the initial soil moisture field is adopted to account for climate change effects in soil moisture given by the GCMs. These climate changes mainly affect the thermodynamics, the lapse rate, and the water budget in the CCLM domain, while the dynamic components in the atmosphere, as well as the model's topography (which would directly affect SMP feedbacks), are left unchanged. Since the climate change signals from the GCMs are spatially averaged in advance, effects of changes in the baroclinicity are being avoided. Note this modelling framework, including the CCLM domain size, has been developed in the underlying study for Maraun et al. (2022).

In this PGW framework, first simulations covering the period 1 October 2008, 00:00 UTC, to 1 October 2009, 00:00 UTC, are conducted for each set of lateral and initial boundary conditions (i.e., IFS with and without imprinted climate change effects). The period before summer 2009, referred to as spin‐up period and excluded from the later analyses, is conducted to balance the jump in resolution (25 to 3 km) in the soil moisture fields. These first simulations are also giving the starting point for the introduction of controlled soil moisture perturbations without destroying the SMP feedbacks of the upper (hydrologic active) soil layers: at the model's initialisation step and during model integration (including spin up), volumetric soil moisture from deep soil layers (below 4 m, that is, layer 9 and 10 in Table 1) is taken from these first (unperturbed) simulations at their storage interval of 1 h, linearly interpolated in time to match the current model time step (a whole‐number multiple of 25 s, see Table 1), and increased/decreased by ±5% and ±10% (referred to as ‘p5pct’, ‘m5pct’, ‘p10pct’, and ‘m10pct’, respectively). To avoid unwanted effects from the time interpolation, additional simulations with 0% changes (regarded to as the reference simulation, ‘ref’) are conducted. (Note a reduction of the storage interval in the soil fields from 1 h to the model's time step of 25 s would drastically slow down the model integration due to the input/output data stream.) In summary, this procedure is equal to a forcing of or a prescribing storyline for deep soil moisture and prevents upper soil layers from unwanted fading effects that would occur if just the initial soil moisture would be perturbed. Since CCLM is fully deterministic and since no additional perturbations are introduced that would account for internal variability (IV) from outside the model domain, like changes in the domain position (e.g., Henneberg et al., 2018) or in the atmospheric initial (e.g., Lavin‐Gullon et al., 2020; Maraun et al., 2022) and lateral boundary conditions (e.g., Giorgi & Bi, 2000), and inside the model domain, as it would be covered in a multi physics ensemble (e.g., Lavin‐Gullon et al., 2020), IV is only covered as it is caused by the introduced deep soil moisture perturbations. It is quantified via the standard deviation (σ) across the perturbed simulations.

These sets of simulations (Table S1) are analysed with respect to (1) deviations from the reference simulations to demonstrate the effects of soil moisture perturbations under current and changing climate conditions and (2) deviations among the sets to investigate climate change effects, both in relation to the perturbation induced IV. The analyses are conducted for the summer season (June, July, August) as well as on an event‐based level. In a preparative step, the volumetric soil moisture θ [m^3^ m^−3^], averaged across the first 6 soil layers, is converted into a dimensionless soil moisture fraction X [−], derived from the wilting point $\theta_{\text{WILT}}$ [m^3^ m^−3^] and the soil saturation $\theta_{\mathrm{SAT}}$ [m^3^ m^−3^], in order to increase comparability among different soil textures and land covers:

(1) $$X=\frac{\theta -\theta_{\text{WILT}}\;}{\theta_{\mathrm{SAT}}-\theta_{\text{WILT}}}$$

The first 6 soil layers cover a total depth of 0.94 m. This encompasses the maximum root depth of 0.7 m that occurs in the modelling domain throughout the entire simulation period. Hence, layer 6 is the last layer that is coupled to the atmosphere via vegetational water uptake (Doms et al., 2013).

Since the modelling domain also covers energy‐limited areas where SMP feedbacks are expected to have a minor impact (Seneviratne et al., 2010; for instance, the mountainous regions), a simple formal criterion is applied in advance to exclude such energy limited areas from further investigations. First, the outermost 30 grid cells of the model domain are removed to avoid unwanted impacts of the lateral sponge zone. Second, the temporal Spearman correlation coefficient between daily evapotranspiration (ET) and daily soil moisture fraction (X) of the period May to September 2009 is calculated in the reference simulation (of the current climate) and smoothed with a thin‐plate‐spline algorithm in order to retrieve a continuous area when applying a threshold of 0.35 in the final step. (Note a simple sensitivity analysis on the selection of this threshold reveals that changing its value to 0.4 or 0.3 changes the extension of the study area by ±4%—an amount that we regard to as neglectable.) This procedure defines the final study area as it is depicted in Figure 1a, b.

In order to indicate systematic deviations in the precipitation fields caused by the introduced soil moisture perturbations, the structure‐amplitude‐location (SAL) index (Wernli et al., 2008) is applied. This index compares all individual precipitation intensity fields for each time step of the stored data of the reference simulation with each corresponding time step of a perturbed soil moisture simulation. The *S*‐component indicates whether the precipitation objects differ in their relation between extension and intensity; the *A*‐component indicates differences in the precipitation amount; the *L*‐component indicates differences in the spatial spread of individual precipitation objects around the centre of precipitated mass. All three components are normalised to achieve comparability. Since the *L*‐component does not provide any information about spatial shifts between corresponding individual precipitation objects in the simulations to be compared, such spatial shifts are additionally calculated as the Euclidian distance between individual centres of mass.

In order to investigate the relationship between summertime precipitation and SMP feedbacks, spatial and temporal SMP coupling strengths ($Y_{s}$ and $Y_{t}$, respectively) as defined by Guillod et al. (2015) based on Taylor, de Jeu, et al. (2012) are correlated with the maximum hourly intensity per individual event (INT). For each individual event, the deviation of the soil moisture fraction (X) from its climatological mean ($\bar{X}$) is calculated 1 h before the event starts and at the locations (Lmin and Lmax) of the event's minimum and maximum intensities. The quantities $Y_{s}$ and $Y_{t}$ are then derived via the following equations:

(2) $$Y_{s}={\left(X-\bar{X}\right)}_{L\max}-{\left(X-\bar{X}\right)}_{L\min}Y_{t}={\left(X-\bar{X}\right)}_{L\max}$$

Since the simulation period does not cover climatological periods, $\bar{X}$ is calculated as running mean in time with a window size of ±15 days. To account for possible diurnal cycles in $\bar{X}$, this procedure is conducted for each hour of the day, separately.

The events are identified as precipitation objects that are connected in space and time with an intensity larger than 0.1 mm h^−1^. The event's spatial and temporal extension that is required to define Lmin, Lmax, and the starting time is defined as its spatial and temporal bounding box in terms of grid cells (plus a buffer zone of one grid cell in each direction) and in terms of the storage interval of the model's data, that is, 1 h (Table 1). After $Y_{s}$, $Y_{t}$, and INT are derived for each individual event, the whole data is statistically analyzed in the following way: First, binned (with a width of 0.01) marginal and bivariate relative frequency distributions for $Y_{s}$ and $Y_{t}$ are calculated. Second, for each bin, the mean INT is estimated and—in order to achieve comparability among all individual simulations including those under changed climate conditions—related to the mean INT across all events. This allows us to investigate the following questions: (1) how are spatial and temporal SMP feedback strengths (that are active prior to the individual precipitation events) distributed and related to each other, (2) how do the SMP coupling strengths relate to precipitation intensities, and (3) is there a climate change impact on the interplay between SMP coupling strengths and precipitation intensities.

## 3 SMP FEEDBACKS UNDER CURRENT CLIMATE CONDITIONS

**FIGURE 2:**
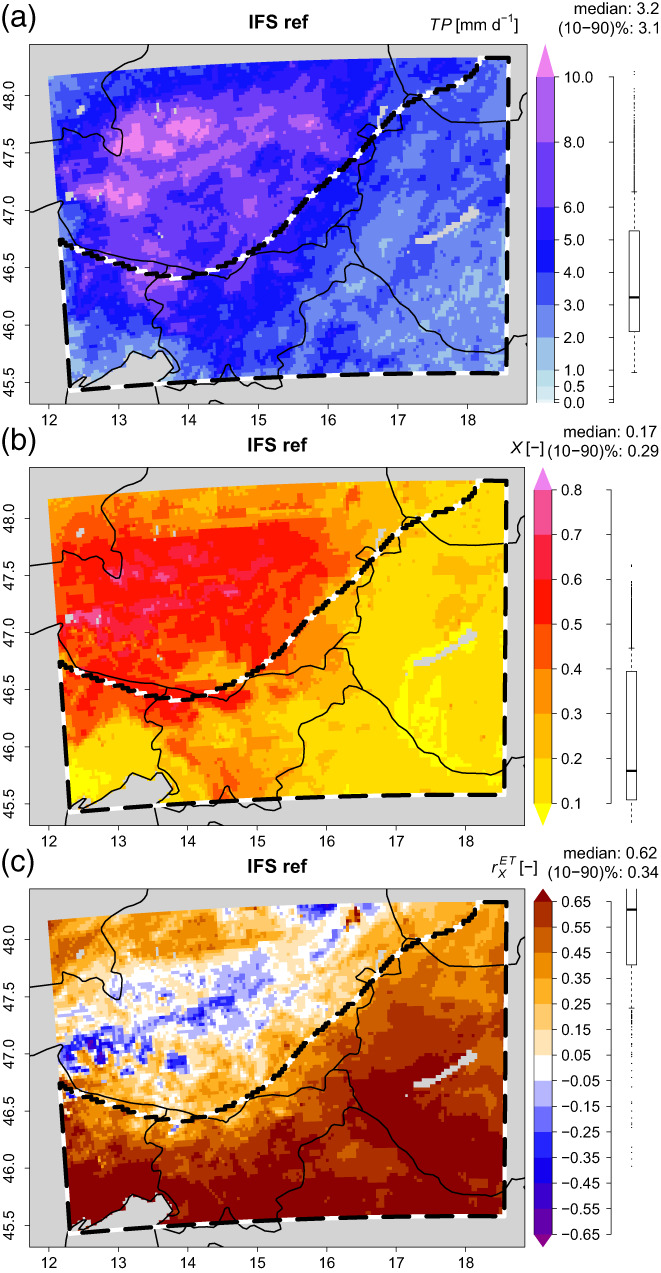
Seasonal (JJA) statistics of the reference simulation driven by IFS. (a) precipitation (TP) [mm day^−1^]; (b) soil moisture fraction (X) [−]; (c) temporal correlation coefficient ($r_{X}^{\mathrm{ET}}$) [−] between daily evapotranspiration (ET) and X from May to September. Distribution, median, and percentile range (‘(10–90)%’) across the study area (b/w dashed polygon) are given to the right of each subfigure. [Colour figure can be viewed at wileyonlinelibrary.com]

The summer season 2009 was rather usual. Apart from an extreme precipitation event from the 22 to 25 June that brought rainfall anomalies of up to 390% in the Eastern Alpine region (Hornich & Adelwöhrer, 2010; ZAMG, 2009a), the rest of the season stayed close to its 30 year average (period 1971–2000) without noticeable heatwaves (ZAMG, 2009b), but still with temperatures about 1–2°C above the average in August (ZAMG, 2009c). In the reference simulation, this gives a seasonal precipitation (TP) of ~3.2 mm day^−1^ (Figure 2a) and a seasonal X of ~0.17 (Figure 2b), averaged (median) across the study area (both increasing when approaching the mountains). The spatial (across the study area) median correlation coefficient between daily actual ET and daily X is 0.62 (Figure 2c) indicating that there is evidence for SMP feedbacks.

**FIGURE 3:**
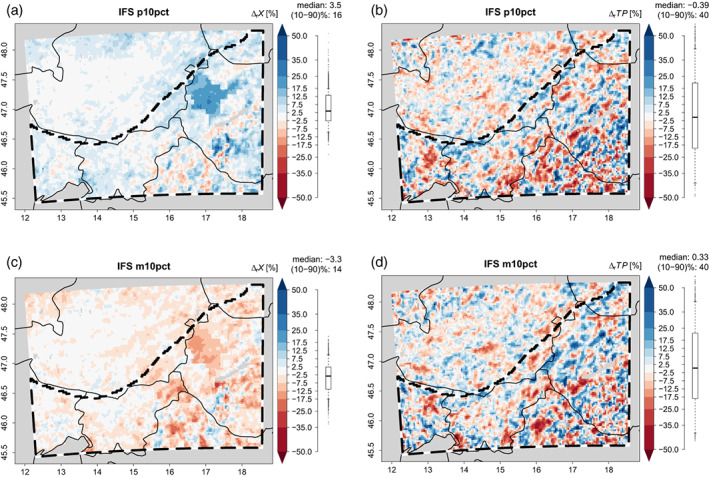
Relative JJA deviations (${∆}_{r}$) [%] of the p10pct (a), (b) and m10pct (c), (d) simulations from the reference simulation with IFS driving data. Deviations in soil moisture fraction (X) (a), (c) and total precipitation (TP) (b), (d) are shown. Distribution (boxplot), median, and percentile range (‘(10–90)%’) across the study area (b/w dashed polygon) are given to the right of each subfigure. [Colour figure can be viewed at wileyonlinelibrary.com]

When the deep soil moisture perturbations are introduced, they cause deviations in the upper layer soil moisture. However, since vertical moisture transport between the upper and deep soil layers is slow, the imprints of the deep soil moisture perturbations in the upper soil layers are dampened and interfere with ET and precipitation which are subject to SMP feedbacks. As a result, the introduced deep soil moisture perturbations lead to local percent relative deviations from the reference simulation in seasonal X with spatial percentile ranges from 13% to 16% (σ=1.3%) and spatial median percent relative deviations within ±3.5% (Table 2), which is about one‐thirdof the introduced deep soil moisture perturbation. σ indicates that the local deviations in X largely exceed IV from deep soil moisture perturbations. In contrast, percent relative local deviations in the seasonal TP are more strongly pronounced: their spatial percentile ranges lie between 41% and 43% and also largely eexceed IV (σ=1.0%), while their spatial median remains nearly unchanged (Table 2)—even if the respective statistical properties of the underlying absolute TP values are nearly identical with those of the reference simulation (Table S2). Figure 3 exemplarily visualises the precent relative local deviations for p10pct and m10pct; the results for p5pct and m5pct are given as supporting information (Figure S1:).

**TABLE 2:** Study area median ($\tilde{∎}$) and percentile range (${∎}_{10}^{90}$) of relative seasonal (JJA) deviations (${∆}_{r}$) [%] of the perturbation simulations from the reference simulation for X and TP (as shown in Figures 3 and S1:) as well as their spatial correlation coefficient ($r_{X}^{\mathrm{TP}}$) and the estimated slope of a linear regression line ($\partial {∆}_{r}\mathrm{TP}/\partial {∆}_{r}X$) under current climate conditions (driving data IFS). The standard deviation (σ) across all perturbed simulations for each column is given in the last row.

Simulation acronym	$\tilde{{∆}_{r}X}$[%]	${\left({∆}_{r}X\right)}_{10}^{90}$[%]	$\tilde{{∆}_{r}\mathrm{TP}}$[%]	${\left({∆}_{r}\mathrm{TP}\right)}_{10}^{90}$[%]	$r_{X}^{\mathrm{TP}}$[−]	$\partial {∆}_{r}\mathrm{TP}/\partial {∆}_{r}X$ [% %^−1^]
p10pct	3.5	16	−0.4	41	0.44	1.3
p5pct	1.9	14	0.3	41	0.54	1.5
m5pct	−1.3	13	0.6	43	0.57	1.8
m10pct	−3.3	14	0.2	41	0.48	1.4
σ	3.1	1.3	0.4	1.0	0.06	0.2

Hence, the introduced deep soil moisture perturbations are too small to cause any noticeable impact on the water budget of the entire domain, but they cause spatial redistributions of the precipitation. On the local scale, deviations in soil moisture of the upper (hydrologically active) soil layers and (to a larger extent) in TP are introduced that are much larger than the induced IV, but with a complex dependency on the sign and magnitude of the deep soil moisture perturbations. There is no scaling or additive relationship between the individual soil moisture perturbation simulations: for instance, the local deviations in TP of p10pct (Figure 3b) are not ‘twice’ as large as those of p5pct (Figure S1b), even if the domain average deep soil moisture increase is doubled (from 5% to 10%).

Since X and TP are coupled and since local deviations in X translate into amplified local deviations in TP in large parts of the study area (see above), a high sensitivity of atmospheric precipitation generating processes from soil moisture perturbations is indicated. Indeed, estimated slopes from linear regression between percent relative seasonal deviations in TP and X lie between 1.3% %^−1^ and 1.8% %^−1^ (without systematic dependency on the sign or magnitude of the deep soil moisture perturbation; Table 2). However, the respective correlation coefficients are rather weak (~0.5, on average). Following Taylor (1990), this indicates that (assuming a linear regression) only about 25% of the local precent relative TP deviations can be explained by local precent relative deviations in X. Hence, (on the seasonal scale) there exists a linear positive SMP feedback, but it accounts for only up to 25% of the local precipitation variability.

Deeper insights into the complex nature of SMP feedbacks in the study area are provided by the event‐based analyses.

**FIGURE 4:**
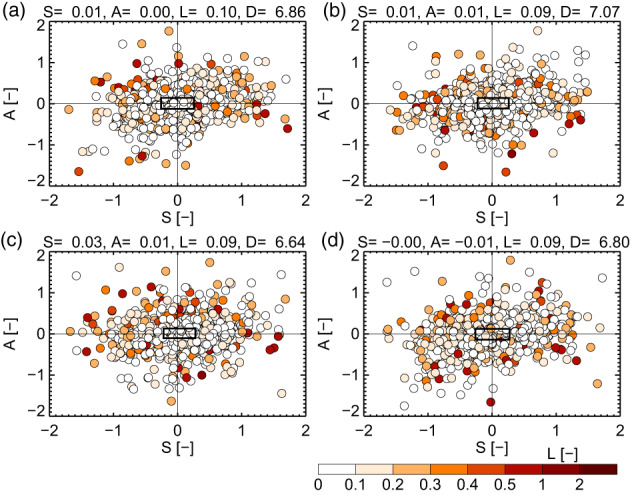
Scatter plot of the structure (S) and amplitude (A) components for JJA from comparing each precipitation field (1330 in total) of (a) p10pct, (b) p5pct, (c) m5pct, (d) m10pct with the reference simulation under current climate conditions (driving data IFS). The location (L) component is given by the colour. The rectangle in the centre indicates the interquartile range (50 % of all data) of S and A. Mean values of S, A, and L are given above each subfigure. D gives the median minimum distance (in multiples of the grid spacing) between corresponding precipitation objects. The standard deviation across the perturbed simulations (σ) of D gives 0.18. [Colour figure can be viewed at wileyonlinelibrary.com]

The SAL statistics reveal that the internal structure of the precipitation fields does not systematically change when the deep soil moisture perturbations are introduced. In all perturbation simulations, the $\left(S,A\right)$ data pairs (1330 in total) are symmetrically distributed and 50% of them are centred on zero in a narrow band (Figure 4). The mean values of S, A, and L components are approximately zero. Such results have also been found by Henneberg et al. (2018) for domain‐averaged soil moisture perturbations smaller than 50% in a specific convective precipitation event in northern Germany.

However, the spatial distances between corresponding precipitation objects in the perturbation simulations and in the reference simulation show that there is an average (median) minimum shift in space of about seven times the grid spacing (i.e., 21 km; Figure 4), largely exceeding IV (σ=0.18, i.e., ~0.5 km). Hence, on average the precipitation objects in the various perturbation simulations may have similar internal characteristics as in the reference simulation, but they are located at different places. Since the only difference between each perturbation simulation and the reference simulation are constant percent relative changes in deep soil moisture (±5%, ±10%), the detected spatial shifts in the location of precipitation objects are nearly exclusively caused by SMP feedbacks (IV due to deep soil moisture perturbations is negligibly small).

**FIGURE 5:**
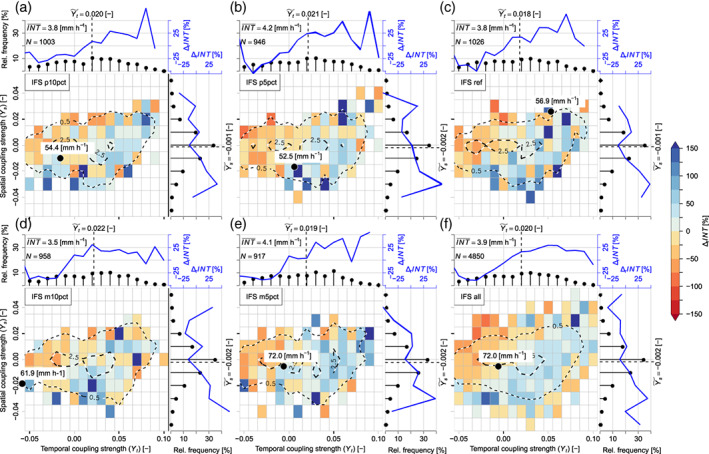
Interplay between spatial and temporal SMP coupling strengths ($Y_{s}$ and $Y_{t}$, respectively) and maximum hourly precipitation intensities (INT) of N isolated precipitation events during JJA in the study area under current climate conditions (driving data: IFS). The colour shaded area depicts estimated intensity anomalies (${∆}_{r}\mathrm{INT}$) from bins (with a width of 0.01) of $Y_{s}$ and $Y_{t}$ related to the given maximum intensity averaged over all events ($\bar{\mathrm{INT}}$). The maximum INT of all events (black dot) is given. The dashed contour lines (levels at 0.5% and 2.5%) are derived from the underlying bivariate percent relative frequency distribution of $Y_{s}$ and $Y_{t}$. Marginal percent relative frequency distributions of $Y_{s}$ and $Y_{t}$ (black dots) together with corresponding percent relative intensity anomalies (blue line) are shown at the top and to the right of each subfigure. Median feedback strengths ($\tilde{Y_{s}}$, $\tilde{Y_{t}}$) are given. (a) simulation p10pct, (b) p5pct, (c) ref, (d) m10pct, (e) m5pct, and (f) all simulations pooled together (referred to as ‘all’). [Colour figure can be viewed at wileyonlinelibrary.com]

The meaning of SMP feedbacks for individual precipitation events becomes obvious when the interplay between the spatial and temporal coupling strengths ($Y_{s}$ and $Y_{t}$ defined in Equation (2)) and their respective precipitation intensities is analysed. As it is shown in Figure 5, $Y_{t}$ is generally broader distributed than $Y_{s}$ and its median is always positive and shows values of about 0.02, regardless of the magnitude or sign of the introduced deep soil moisture perturbation. The median of $Y_{s}$, however, is much smaller and has no systematic sign. (Note, due to the large number of events, the medians of both, $Y_{t}$ and $Y_{s}$, differ significantly from zero.) Hence, more precipitation events are associated with a positive temporal SMP coupling than with a negative one. However, heavy and severe precipitation events with maximum intensities above the mean (blueish shaded area in Figure 5) are in favour of either positive temporal combined with positive spatial coupling or negative spatial coupling combined with temporal coupling around zero. In contrast, light precipitation events (maximum intensities below the mean; reddish shaded area in Figure 5) are in favour of negative temporal combined with positive spatial coupling. This general behaviour is found in all simulations and is hence independent from IV. Moreover, the soil moisture perturbations have no systematic impact on the averaged maximum intensity—values range from 3.5 mm h^−1^ in m10pct (Figure 5d) to 4.2 mm h^−1^ in p5pct (Figure 5b). Hence, when all simulations are pooled (in order to increase the number of events to 4850), the interplay between the SMP coupling strengths and the precipitation intensities becomes even more clear (Figure 5f).

## 4 SMP FEEDBACKS UNDER FUTURE CLIMATE CONDITIONS

**FIGURE 6:**
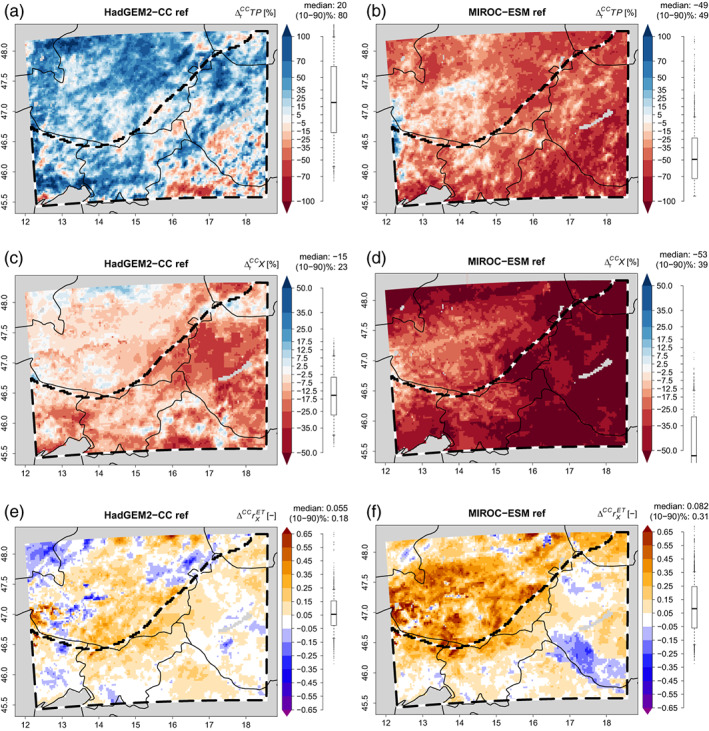
Seasonal (JJA) climate change effects in the reference simulation when climate changes from HadGEM2‐CC (a), (c), (e) and MIROC‐ESM (b), (d), (f) are applied. (a), (b)Relative precipitation changes (${∆}_{r}^{\mathrm{CC}}\mathrm{TP}$) [%]; (c), (d) relative soil moisture fraction changes (${∆}_{r}^{\mathrm{CC}}X$) [%]; (e), (f) absolute changes in the temporal correlation coefficient (${{∆}^{\mathrm{CC}}r}_{X}^{\mathrm{ET}}$) [−] between daily evapotranspiration (ET) and X from May to September. Distribution, median, and percentile range (‘(10–90)%’) across the study area (b/w dashed polygon) are given to the right of each subfigure. [Colour figure can be viewed at wileyonlinelibrary.com]

When climate change effects (periods 2071–2100 vs. 1985–2005; RCP 8.5) from the various GCMs are imprinted, CCLM gives divergent climate change signals: as it is shown in Table 3, HadGEM‐CC lets summertime TP increase by about 20% and X decrease by 15% across the study area, while IPSL‐CM5A‐MR and MIROC‐ESM give decreases in TP on the order of 50% together with strong reductions in X of about 53%. GFDL‐ESM2M gives −42% in X and −45% in TP. The spatial distributions of such diverting climate change conditions are exemplarily depicted for HadGEM2‐CC and MIROC‐ESM in Figure 6 (the results for IPSL‐CM5A‐MR and GFDL‐ESM2M are shown in the supporting information Figure S2). Although the spatial averaged correlation between ET and X is increased throughout all the GCMs (Table 3), indicating that the SMP feedbacks become generally stronger, Figures 6e, f, S2e, and S2f show that there are subareas of a decreasing correlation. In these subareas, the soil moisture approaches the wilting point and the availability of moisture is strongly reduced. Hence, the energy from net solar radiation is turned into sensible instead of latent heat. As a consequence, ET becomes approximately zero and the correlation with X gets lost. This happens more often under dryer conditions, like MIROC‐ESM (Figure 6f), IPSL‐CM5A‐MR (Figure S2e), and GFDL‐ESM2M (Figure S2f) than under increased humid conditions, like HadGEM2‐CC (Figure 6e). Note, in all changed climate conditions the correlation between ET and X increases even stronger in energy‐limited mountainous areas, indicating that SMP feedbacks become more important in elevated areas under future climate conditions.

**TABLE 3:** Study area median ($\tilde{∎}$) and percentile range (${∎}_{10}^{90}$) of relative [%] climate change effects (${∆}_{r}^{\mathrm{CC}}$) in seasonal (JJA) soil moisture fraction (X), precipitation (TP) (as depicted in Figures 6a–d and S2a–d), and the temporal correlation coefficient ($r_{X}^{\mathrm{ET}}$) between X and evapotranspiration (ET) from May to September in the reference simulations with thermodynamic climate changes from the given GCMs.

CGM	$\tilde{{∆}_{r}^{\mathrm{CC}}X}$[%]	${\left({∆}_{r}^{\mathrm{CC}}X\right)}_{10}^{90}$[%]	$\tilde{{∆}_{r}^{\mathrm{CC}}\mathrm{TP}}$[%]	${\left({∆}_{r}^{\mathrm{CC}}\mathrm{TP}\right)}_{10}^{90}$[%]	$\tilde{{{∆}^{\mathrm{CC}}r}_{X}^{\mathrm{ET}}}$ [−]	${\left({{∆}^{\mathrm{CC}}r}_{X}^{\mathrm{ET}}\right)}_{10}^{90}$[−]
HadGEM2‐CC	15.2	22.9	20	82	0.06	0.18
IPSL‐CM5A‐MR	−53.5	41.1	−52	45	0.08	0.27
MIROC‐ESM	−53.2	38.7	−50	50	0.08	0.31
GFDL‐ESM2M	−42.1	34.8	−45	49	0.09	0.26

Implementing the deep soil moisture perturbations into the PGW framework reveals that spatial median percent relative deviations from the reference simulation in X between −4.5% and 5.1% (Table 4) together with spatial percentile range from 11% to 17% (Table 4) are caused. These are of similar magnitudes as under current climate conditions (Table 2), even if σ is slightly increased. The spatial median of local percent relative deviations in TP is again very small (<1.5%, Table 4), but the underlying spatial percentile ranges are larger than under current climate conditions (Table 2) and lie between 49% and 68% with still small σ (Table 4). The spatial distribution of local deviations in X and TP for the p10pct and m10pct simulations of all GCMs can be found in Figures S3–S6. Hence, the sensitivity of atmospheric precipitation generating processes to soil moisture perturbations is increased under the implemented changed climate conditions. However, the slopes of linear regressions between local percent relative deviations in TP and X are broader distributed than under current climate (Table 2) and range from 0.4% %^−1^ (HadGEM‐CC) to 1.9% %^−1^ (GFDL‐ESM2M) and the correlation coefficients are much weaker (~0.25, on average), regardless of the underlying GCM (Table 4). Hence, under any of the introduced climate changes, there exists a positive SMP feedback on the seasonal scale, but it is more uncertain than under current climate conditions and accounts for only up to 6.25% of the local seasonal TP variability.

**TABLE 4:** Study area median ($\tilde{∎}$) and percentile range (${∎}_{10}^{90}$) of relative seasonal (JJA) deviations [%] from the reference simulation of soil moisture fraction (X) and precipitation (TP) as well as their spatial correlation coefficient ($r_{X}^{\mathrm{TP}}$) and the estimated slope of the linear regression line (∂TP/∂X), caused by thermodynamic changes from given GCMs. The standard deviation (σ) across the perturbed simulations for each GCM is given in separate intermediate rows.

GCM	Simulation acronym	$\tilde{X}$[%]	$X_{10}^{90}$[%]	$\tilde{\mathrm{TP}}$[%]	${\mathrm{TP}}_{10}^{90}$[%]	$r_{X}^{\mathrm{TP}}$[−]	∂TP/∂X [% %^−1^]
HadGEM2‐CC	p10pct	4.1	17	1.3	49	0.19	0.4
HadGEM2‐CC	p5pct	2.0	14	0.6	49	0.32	0.9
HadGEM2‐CC	m5pct	−1.4	13	1.1	49	0.21	0.6
HadGEM2‐CC	m10pct	−2.4	15	1.0	49	0.21	0.5
	σ	3.0	1.7	0.3	0.0	0.06	0.2
IPSL‐CM5A‐MR	p10pct	4.3	14	1.6	62	0.24	1.1
IPSL‐CM5A‐MR	p5pct	1.6	11	0.2	63	0.31	1.5
IPSL‐CM5A‐MR	m5pct	−1.3	11	1.5	66	0.33	1.7
IPSL‐CM5A‐MR	m10pct	−2.5	12	1.3	60	0.31	1.6
	σ	3.0	1.4	0.6	2.5	0.04	0.3
MIROC‐ESM	p10pct	4.7	16	−1.1	68	0.17	0.8
MIROC‐ESM	p5pct	2.2	13	−1.0	67	0.17	1.2
MIROC‐ESM	m5pct	−2.5	13	−0.8	67	0.21	1.6
MIROC‐ESM	m10pct	−4.5	14	−1.5	68	0.20	1.3
	σ	4.2	1.4	0.3	0.6	0.02	0.3
GFDL‐ESM2M	p10pct	5.1	17	0.8	59	0.23	0.7
GFDL‐ESM2M	p5pct	2.3	11	−1.3	57	0.28	1.1
GFDL‐ESM2M	m5pct	−2.1	11	0.5	59	0.33	1.9
GFDL‐ESM2M	m10pct	−3.8	14	−0.8	59	0.23	0.9
	σ	4.1	2.9	1.0	1.0	0.05	0.5

**FIGURE 7:**
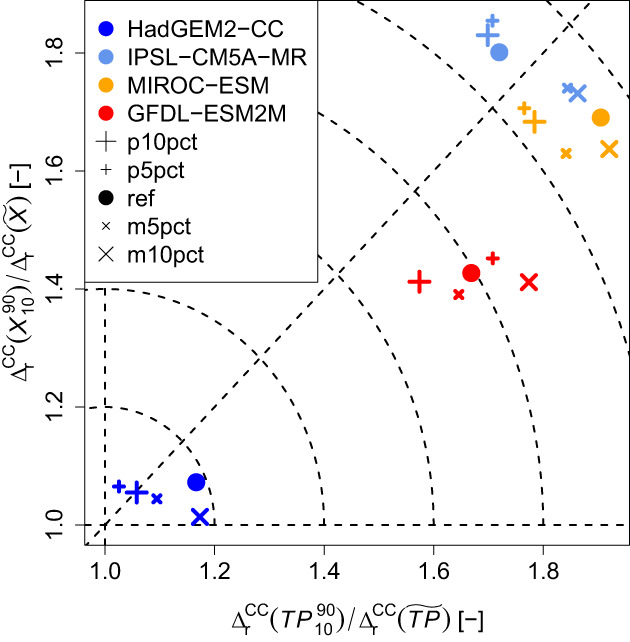
Ratios of relative climate change signals (${∆}_{r}^{\mathrm{CC}}$) of spatial percentile range (${∎}_{10}^{90}$) and spatial median ($\tilde{∎}$) in the study area of seasonal (JJA) soil moisture fraction (X) and precipitation (TP), caused by thermodynamic changes from various GCMs (coloured symbols). Perturbation simulations and the reference simulation per climate change are indicated by individual symbols of the same colour. [Colour figure can be viewed at wileyonlinelibrary.com]

On the other hand, TP and X are directly affected by climate change. Since in this PGW framework climate change is mainly dominated by thermodynamic changes and the actual study area is rather homogeneous and fairly departed from orographic influences of the Eastern Alps, one would expect to find constant fractional changes (Pfahl et al., 2017). Hence, it is reasonable to assume, that if domain averages of TP and X are brought onto other levels via climate change, their underlying spatial anomalies will scale with the averaged climate change signals. For instance, if TP and X drop by 50% due to climate change, one would assume that also their spatial anomalies would drop by 50%. Assuming such a simple scaling, the ratios between the relative climate change signals of the spatial percentile ranges and the relative climate change signals of the spatial medians (i.e., ratios of two ratios) of TP and X of all (perturbation and reference) simulations would lie close to 1. However, as it can be seen in Figure 7, only the HadGEM2‐CC case gives changes in TP and X that are approximately in line with this linear scaling. The other GCMs (characterised by strong decreases in TP and X of about 50%; cf., Table 3) lead to larger reductions in the relative climate change signals of the spatial medians of TP and X than in their respective relative climate change signals of the spatial percentile ranges (their ratios are obviously larger than 1; cf., Figure 7). In other words, the spatial percentile ranges of TP and X remain larger than expected from linear climate change scaling. Since spatial heterogeneity in soil moisture has been found to be an important factor for SMP feedbacks (Guillod et al., 2015; Taylor et al., 2011), such ratios larger than 1 indicate that the influence of SMP feedbacks becomes stronger than expected from mean climate change. The perturbed simulations do not introduce noteworthy systematics—the impact of their respective IV is minor.

When the climate changes are applied, the SAL statistics (Figures S7–S10) give similar results as under current climate conditions (Figure 4): systematic changes in the internal structure of the precipitation objects are not indicated. All the $\left(S,A\right)$ data pairs are symmetrically distributed, 50% of them are centred around zero in a narrow band, and the mean values of the S, A, and L components are approximately zero (Figures S7–S10). Also, the spatial distances between corresponding precipitation objects in the perturbation simulations and the reference simulations indicate that there is a shift in space of about 7–8 times the grid spacing (i.e., 21–24 km) (the larger shifts are found for HadGEM2‐CC) that is independent of the introduced soil moisture perturbations (Figures S7–S10). Again, IV has a minor impact and there is no scaling or additive relation among the soil moisture perturbation simulations in the SAL statistics.

**FIGURE 8:**
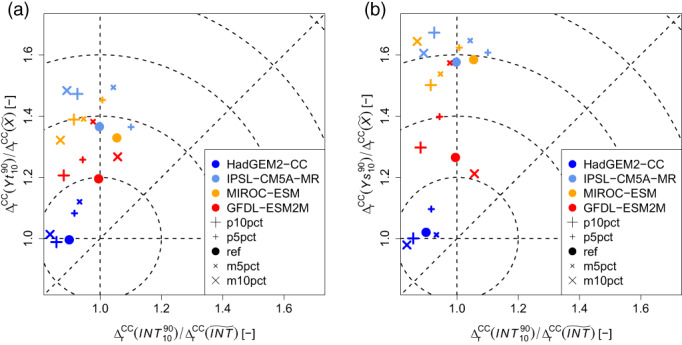
Ratios of relative climate change signals (${∆}_{r}^{\mathrm{CC}}$) of percentile range (${∎}_{10}^{90}$) and median ($\tilde{∎}$) of seasonal (JJA) soil moisture fraction (X), event related maximum precipitation intensities (INT), and (a) temporal and (b) spatial SMP feedback strengths ($Y_{t}$, $Y_{s}$), caused by climate changes from various GCMs (coloured symbols). Perturbation simulations and the reference simulation per climate change are indicated by individual symbols of the same colour. [Colour figure can be viewed at wileyonlinelibrary.com]

The analyses of SMP feedbacks on the basis of individual precipitation events show that the interplay between the coupling strengths and the corresponding maximum precipitation intensities does not fundamentally change when the various climate change effects are applied and the soil moisture perturbations are introduced. As it is shown in Figures S11–S14, individual precipitation events may coincide with all types and all signs of coupling strengths, however, heavy and severe precipitation events (blueish shaded areas) are in favour of either a positive temporal combined with a positive spatial coupling or a negative spatial coupling combined with a temporal coupling around zero. In contrast, light precipitation events (reddish shaded areas) are in favour of a negative temporal combined with a positive spatial coupling. This qualitative interplay is also found under current climate conditions (Figure 5), but when the changing climate conditions are introduced the distributions of $Y_{s}$ and $Y_{t}$ become generally narrower. This happens because X is reduced due to climate change (Table 3) and $Y_{s}$ and $Y_{t}$ are defined as deviations from the climatological mean of X (cf., Equation (2)). On the other hand, the precipitation intensities per event are also directly affected by climate change. Following Pfahl et al. (2017), one would expect to find constant scaling factors between the climate change fractions of individual precipitation intensities (and $Y_{s}$ and $Y_{t}$) and their spatial average change, if climate change is dominated by thermodynamic changes. As it can be seen in Figure 8, such a linear scaling is approximately found for the event maximum precipitation intensities (the ratios lie close to 1 within a narrow band from 0.8 to 1.2), regardless of both, the applied changing climate conditions and the soil moisture perturbations. Only in the HadGEM2‐CC case, $Y_{t}$ (Figure 8a) and $Y_{s}$ (Figure 8b) scale linearly with seasonal climate change in soil moisture (the ratios range from 0.95 to 1.15). In the other cases, $Y_{t}$ and $Y_{s}$ lie well beyond linear scaling, especially $Y_{s}$ gains systematically higher ratios (up to 1.7) than $Y_{t}$ (up to 1.5). The soil moisture perturbations do not show any systematic impact, indicating that the influence of IV is minor. Hence, when climate conditions become strongly dryer, the SMP coupling (temporal and spatial, positive and negative) becomes stronger, because climate change scaling needs to be considered in the interpretation of the calculated coupling strengths.

## 5 IMPACT OF SOIL MOISTURE UNCERTAINTY ON CLIMATE CHANGE SIGNALS OF SEASONAL PRECIPITATION

The introduced perturbations in deep soil moisture are constant percentages ranging from −10% to +10%. Here, they are being interpreted as systematic biases in the representation of deep soil moisture. Such biases may occur due to simplifications or shortcomings in the modelling approach of soil processes. As such, they represent a source of uncertainty.

In this section, the effects of various combinations of deep soil moisture perturbations on the climate change signals for seasonal summertime precipitation in the study area are analysed. More specifically, this section tackles the question whether divergent deep soil moisture perturbations under current and changed climate conditions give different climate change signals than if the perturbations are homogeneous or the same. For instance, does the climate change signal calculated from the perturbed simulation p10pct of the current climate and the simulation m10pct of the changed climate differ from the climate change signal of simulation p10pct under current and changed climate conditions?

**FIGURE 9:**
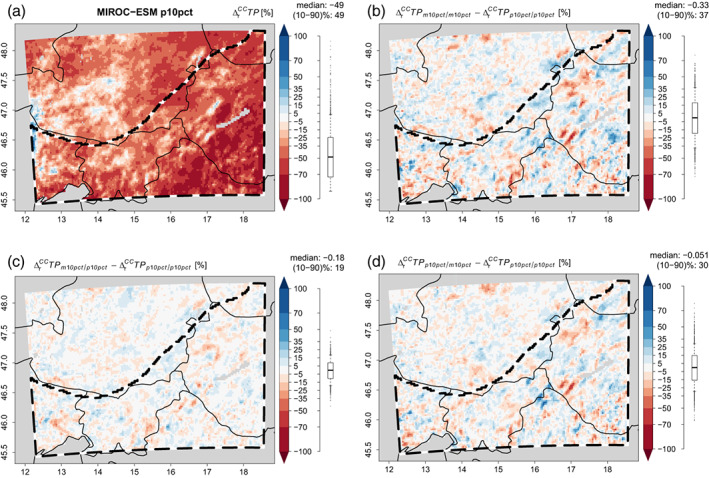
Seasonal (JJA) climate change effects for precipitation (${∆}_{r}^{\mathrm{CC}}\mathrm{TP}$) [%] from (a) the p10pct simulation in current and changed climate based on MIROC‐ESM. (b) differences in ${∆}_{r}^{\mathrm{CC}}\mathrm{TP}$ when m10pct is used in current and changed climate, instead of p10pct; (c) differences in ${∆}_{r}^{\mathrm{CC}}\mathrm{TP}$ when m10pct is used in current climate, only; (d) differences in ${∆}_{r}^{\mathrm{CC}}\mathrm{TP}$ when m10pct is used in changed climate, only. Distribution, median, and percentile range (‘(10–90)%’) across the study area (b/w dashed polygon) are given to the right of each subfigure. [Colour figure can be viewed at wileyonlinelibrary.com]

The answer to this question is exemplarily shown for the MIRCO‐ESM case in Figure 9. p10pct in current and changed climate reduces summertime precipitation by 49% (spatial median) with individual fluctuations in a spatial percentile range of 49% (Figure 9a). When changing to m10pct in the current and the changed climate, local deviations in the percent relative climate change signal with a spatial percentile range of 37% are introduced (the spatial median change is negligibly affected) (Figure 9b). Changing the perturbated simulation to m10pct in the changed climate conditions only, introduces local deviations in the climate change signal with a spatial percentile range of 30% while the spatial median of the deviations is again rather unaffected (−0.05%) (Figure 9d). In addition, the spatial pattern in the changes of the climate change signal matches well with the pattern caused by changing to the pure m10pct case (compare Figure 9b with Figure 9d). Changing the perturbation simulation to m10pct for the current climate conditions only (Figure 9c), has a similar effect—however, it is less pronounced (the spatial percentile range of the local deviations in the climate change signal is merely 19 %) and shows an uncorrelated pattern (Figure 9c). Hence, largest impacts of uncertainty in deep soil moisture are found when homogenously switching from one perturbation to the next in both, the current and future climate conditions. However, these impacts are dominated by divergent deep soil moisture uncertainty in future climate conditions.

**FIGURE 10:**
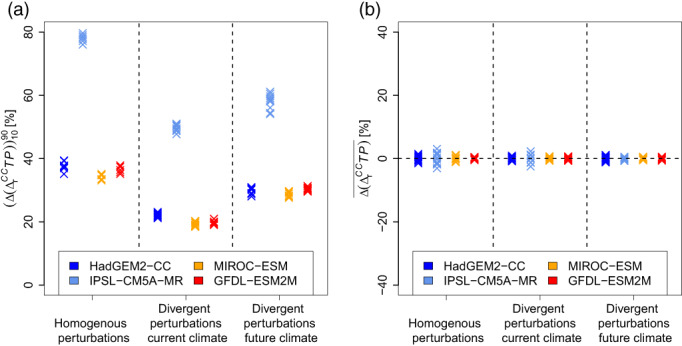
Differences in relative climate change signals of seasonal summertime precipitation (${∆}_{r}^{\mathrm{CC}}\mathrm{TP}$) between different combinations of deep soil moisture perturbations under various climate change conditions (coloured marks). Homogenous perturbations in current and future climate, divergent perturbations in current combined with homogenous perturbations in future climate, and divergent perturbations in future combined with homogenous perturbations in current climate are shown. (a) percentile range (${∎}_{10}^{90}$) in the study area; (b) spatial mean value ($\bar{∎}$). [Colour figure can be viewed at wileyonlinelibrary.com]

The results of an extended analysis that includes all possible combinations of deep soil moisture perturbations in the calculation of the climate change signal are shown in Figure 10. Under all climate change conditions, local deviations in the climate change signals are most pronounced when deep soil moisture perturbations are homogenously applied in both, the current and the future climate conditions. In this case, the local deviations show spatial percentile ranges between 30 % and 80 % (Figure 10a). In other words, uncertainty in deep soil moisture perturbations in the order of ±10% are causing local deviations in the relative seasonal climate change signals of precipitation from ±15% to ±40% (spatial half percentile ranges). They become weaker (spatial percentile ranges between 25% and 60%) when the perturbations divert only under future climate conditions. When the perturbations divert only under current climate conditions, the local changes in the climate change signals are weakest (spatial percentile ranges between 18% and 50%). Since the spreads of data points within individual GCMs in Figure 10a are obviously smaller than the differences induced by the differences in the calculation of the respective climate change signals, the impact of IV is minor. The spatial median of the deviations in all combinations of deep soil moisture perturbations and in all changing climate conditions is again unaffected and lies between ±3% (Figure 10b). This confirms the results found for the MIROC‐ESM case and underpins the hypothesis that the impact of uncertainty of deep soil moisture on the climate change signal is more important for future than for current climate conditions.

## 6 CONCLUSIONS

In the presented study, a novel modelling framework that combines the advantages of the PGW approach and convection‐permitting models and allows to investigate soil moisture atmosphere feedbacks via prescribed deep soil moisture storylines is introduced. The RCM CCLM (3 km grid spacing; without deep convection parameterisation) is operated in a moisture‐limited study area that ranges from the Eastern Alpine foothills to the western Pannonian Basin. Perturbations of deep soil moisture ranging from ±5% to ±10% are introduced to investigate the sensitivity of summertime precipitation to SMP feedbacks. In addition, opposing (more humid vs. severely dryer) climate change effects from four GCMs (HadGEM2‐CC, IPSL‐CM5A‐MR, MIROC‐ESM, and GFDL‐ESM2M) employing the RCP 8.5 scenario are applied onto CCLM's driving data in a PGW framework. This enables us to elaborate how SMP feedbacks change under changing climate conditions and how uncertainty in deep soil moisture may affect climate change signals.

From the applied analyses, the function of SMP feedbacks in the study area can be summarised as follows. All precipitation events are affected by SMP feedbacks. However, heavy and severe events can be attributed to one of the two combinations: (1) positive temporal and positive spatial SMP coupling or (2) negative spatial and almost no temporal coupling. In contrast, light precipitation events are in favour of negative temporal combined with positive spatial SMP coupling. The soil moisture field at a certain point in time (which depends on previous precipitation events via the soil's memory, aggregated ET, and vertical moisture transport) and particularly its spatial variability strongly affects the location of individual upcoming precipitation objects (a median minimum shift of seven times the grid spacing, i.e., 21 km, has been found), while the internal structure of precipitation objects does not systematically change. In particular, the amount of soil moisture perturbations averaged across the study area has a negligible impact on both, the seasonal precipitation amount and the averaged maximum precipitation intensity per event. Consequently, the analyses suggest that the soil moisture field acts as a guiding field for the position of the next precipitation event by means of temporal and spatial SMP coupling.

Aggregated over the entire summer season, this complex interplay between temporal and spatial SMP coupling and precipitation intensities that is active for each single precipitation event leads to an overall positive seasonal SMP feedback. But only 25% of the local seasonal precipitation variability can be explained by a linear regression between local seasonal soil moisture and seasonal precipitation anomalies.

Under any of the investigated climate change conditions, the fundamental role of SMP feedbacks does not change. The introduced soil moisture perturbations do not systematically affect the internal structure of precipitation objects but cause spatial shifts in the order of 7–8 times the grid spacing, that is, 21–24 km. Hence, the role of the soil moisture for guiding the next precipitation event is independent of thermodynamic climate change. Again, heavy and severe events are more likely under either positive temporal and positive spatial coupling or negative spatial and almost no temporal couplings, while light precipitation events are in preference of negative temporal combined with positive spatial coupling. However, drying climate conditions that give strong reductions in both, seasonal (summer) soil moisture and precipitation in the order of 50% (derived from IPSL‐CM5A‐MR, MIROC‐ESM, and GFDL‐ESM2M), show an enhanced increase in the SMP coupling strengths (spatial more than temporal) that is 1.5–1.7 times larger than expected from linear climate change scaling. Nonetheless, such a linear scaling of SMP coupling strengths with climate change has been found under more humid climate conditions (derived from HadGEM2‐CC) that lets seasonal summer precipitation increase by 20% and lets seasonal soil moisture moderately decrease by 15%.

The increase of the importance of SMP feedbacks in all climate change conditions is also reflected by the correlation coefficient between daily ET and soil moisture fraction: it experiences further minor increases. Strongest increases of up to 0.55, however, are found even outside of the study area, like in the Basin of Graz and in the mountainous regions of the Eastern Alps, indicating a further extension of moisture limited areas due to thermodynamic climate change.

A first analysis on the impact of deep soil moisture perturbations on the calculation of the climate change signal of seasonal summertime precipitation reveals a strong sensitivity with respect to the signal's spatial distribution. Local deviations in the climate change signal that is specifically dominated by changes in the deep soil moisture perturbation in future climate conditions are introduced and lie within spatial half percentile ranges from ±15% to ±40% (depending on the imprinted climate change), while domain‐averaged deviations are negligibly small. This underpins the vital importance of an accurate and physically correct representation of deep soil moisture (particularly under future climate conditions) when local climate change effects are of concern.

Although the derived results and drawn conclusions are purely based on synoptic conditions from October 2008 to (including) September 2009, they may also be valid for other periods: on one hand, the summer season 2009 was rather usual and on the other hand, the role of SMP feedbacks has been found to be robust against strong and opposing thermodynamic climate change effects and perturbations in deep soil moisture and their respectively induced IV. Hence, also IV from outside the model domain would not have a relevant impact as long as it does not include drastic changes in the synoptic conditions. However, small‐scale IV from turbulence and micro physics is not properly captured in this study and hence, the choice of the model (CCLM, in this case) might have a crucial impact. Since model‐specific deviations in land surface and atmospheric parameterisations are found to be important factors for SMP feedbacks (e.g., Knist et al., 2017) and model IV (e.g., Lavin‐Gullon et al., 2020), other models than CCLM could give significantly different results with respect to the interplay between SMP coupling strengths and precipitation intensities, the SMP feedbacks’ thermodynamic climate change scaling behaviour, and the impact of deep soil moisture uncertainty on climate change signals. In order to overcome such limitations, multi‐model multi‐physics ensemble simulations should be conducted within this novel PGW/soil moisture storyline modelling framework.

## AUTHOR CONTRIBUTIONS

**Heimo Truhetz:** Conceptualization; investigation; funding acquisition; writing – original draft; visualization; formal analysis; methodology; writing – review and editing. **Aditya N. Mishra:** Conceptualization; methodology; writing – original draft; writing – review and editing.

## Supporting information


**Figure S1:** Relative deviations of p5pct and m5pct from the reference simulation.
**Figure S2:** Seasonal climate change effects in the reference simulation under IPSL‐CM5A‐MR and GFDL‐ESM2M climate conditions.
**Figure S3:** Relative deviations of p10pct and m10pct from the reference simulation under HadGEM2‐CC climate conditions.
**Figure S4:** Relative deviations of p10pct and m10pct from the reference simulation under IPSL‐CM5A‐MR climate conditions.
**Figure S5:** Relative deviations of p10pct and m10pct from the reference simulation under MIROC‐ESM climate conditions.
**Figure S6:** Relative deviations of p10pct and m10pct from the reference simulation under GFDL‐ESM2M climate conditions.
**Figure S7:** SAL‐statistics under HadGEM2‐CC climate conditions.
**Figure S8:** SAL‐statistics under IPSL‐CM5A‐MR climate conditions.
**Figure S9:** SAL‐statistics under MIROC‐ESM climate conditions.
**Figure S10:** SAL‐statistics under GFDL‐ESM2M climate conditions.
**Figure S11:** Interplay between spatial and temporal SMP coupling strengths and maximum hourly precipitation intensities under HadGEM2‐CC climate conditions.
**Figure S12:** Interplay between spatial and temporal SMP coupling strengths and maximum hourly precipitation intensities under IPSL‐CM5A‐MR climate conditions.
**Figure S13:** Interplay between spatial and temporal SMP coupling strengths and maximum hourly precipitation intensities under MIROC‐ESM climate conditions.
**Figure S14:** Interplay between spatial and temporal SMP feedback strengths and maximum hourly precipitation intensities under GFDL‐ESM2M climate conditions.
**Table S1:** Table of conducted simulations.
**Table S2:** Spatial statistics of the perturbed simulations under current climate conditions.
