# Supplementary material for: Soil moisture precipitation feedbacks in the Eastern European Alpine region in convection‐permitting climate simulations
Source: Int J Climatol. 2023 Sep 10;43(14):6763–82. doi: 10.1002/joc.8234 (PMC10947590; doi:10.1002/joc.8234)
Supplement: Supplementary file 1 — Figure S1: Relative deviations of p5pct and m5pct from the reference simulation. Figure S2: Seasonal climate change effects in the reference simulation under IPSL‐CM5A‐MR and GFDL‐ESM2M climate conditions. Figure S3: Relative deviations of p10pct and m10pct from the reference simulation under HadGEM2‐CC climate conditions. Figure S4: Relative deviations of p10pct and m10pct from the reference simulation under IPSL‐CM5A‐MR climate conditions. Figure S5: Relative deviations of p10pct and m10pct from the reference simulation under MIROC‐ESM climate conditions. Figure S6: Relative deviations of p10pct and m10pct from the reference simulation under GFDL‐ESM2M climate conditions. Figure S7: SAL‐statistics under HadGEM2‐CC climate conditions. Figure S8: SAL‐statistics under IPSL‐CM5A‐MR climate conditions. Figure S9: SAL‐statistics under MIROC‐ESM climate conditions. Figure S10: SAL‐statistics under GFDL‐ESM2M climate conditions. Figure S11: Interplay between spatial and temporal SMP coupling strengths and maximum hourly precipitation intensities under HadGEM2‐CC climate conditions. Figure S12: Interplay between spatial and temporal SMP coupling strengths and maximum hourly precipitation intensities under IPSL‐CM5A‐MR climate conditions. Figure S13: Interplay between spatial and temporal SMP coupling strengths and maximum hourly precipitation intensities under MIROC‐ESM climate conditions. Figure S14: Interplay between spatial and temporal SMP feedback strengths and maximum hourly precipitation intensities under GFDL‐ESM2M climate conditions. Table S1: Table of conducted simulations. Table S2: Spatial statistics of the perturbed simulations under current climate conditions. [file JOC-43-6763-s001.docx]

Supporting information

Soil moisture precipitation feedbacks in the Eastern European Alpine region in convection permitting climate simulations

Heimo Truhetz^1^ and Aditya N. Mishra^1^

^1^Wegener Center for Climate and Global Change (WEGC), University of Graz, Graz, Austria

**Correspondence**

Heimo Truhetz, University of Graz, Wegener Center for Climate and Global Change (WEGC), Brandhofgasse 5, A-8010 Graz, Austria. Email: [heimo.truhetz@uni-graz.at](mailto:heimo.truhetz@uni-graz.at)

# Figures


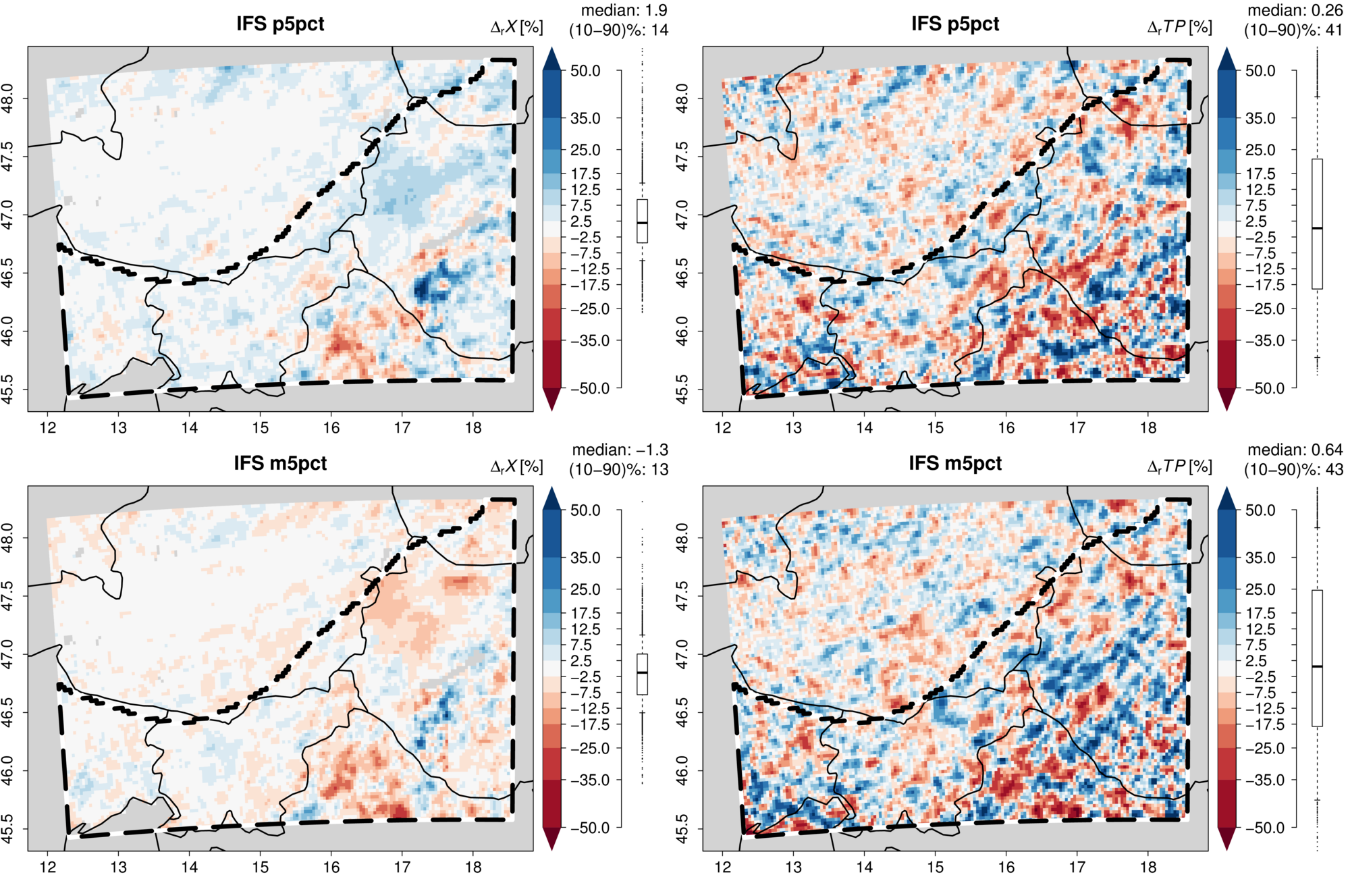


(a)

(c)

(b)

(d)

**Figure S1** Relative JJA deviations ($\Delta_{r}$) [%] of the (a, b) p5pct and (c, d) m5pct simulations from the reference simulation with IFS driving data. (a, c) Deviations in soil moisture fraction ($X$) and (b, d) total precipitation ($TP$) are shown. Distribution (boxplot), median, and percentile range (“(10-90)%”) across the study area (b/w dashed polygon) are given to the right of each subfigure.


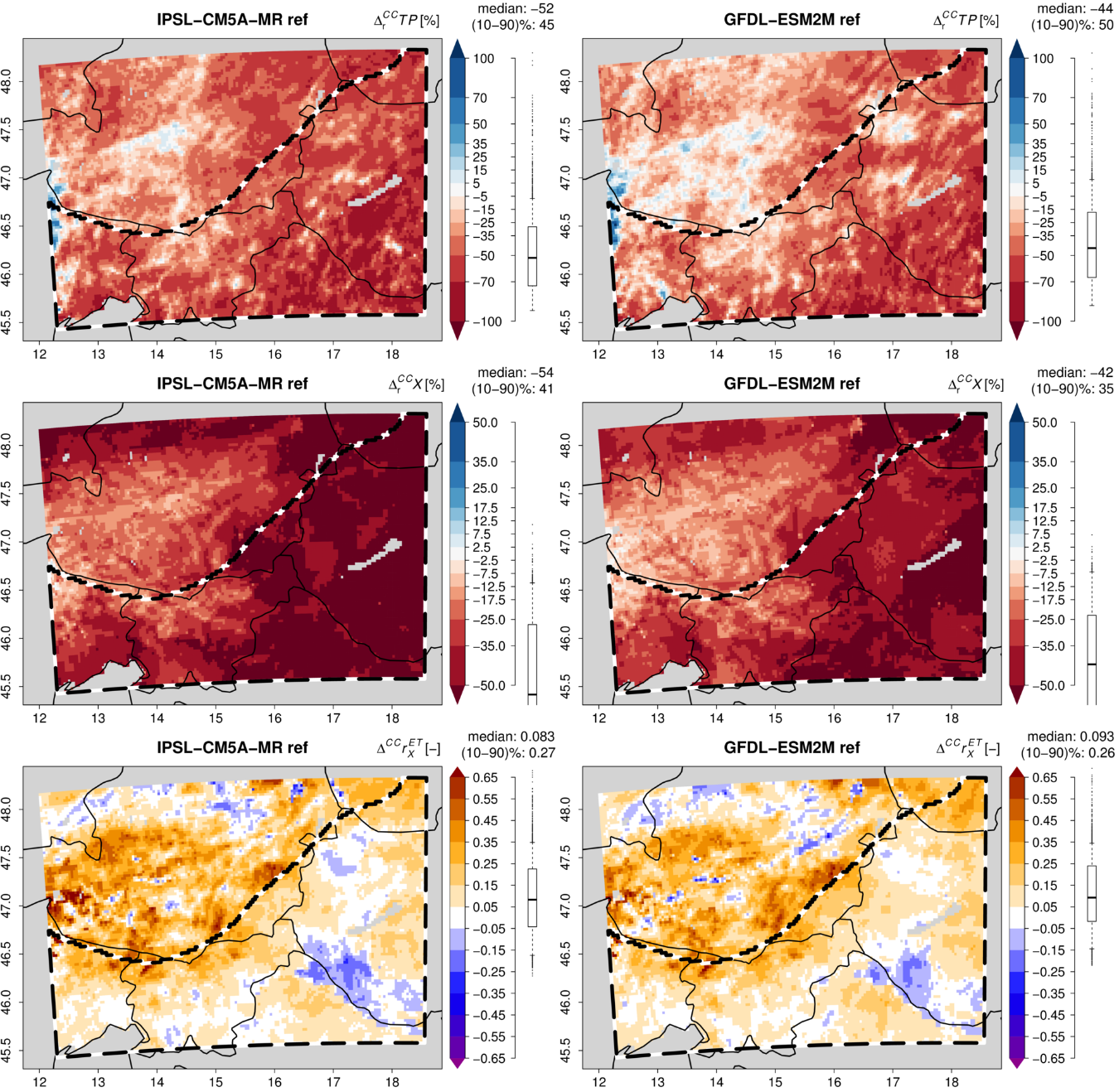


(f)

(e)

(c)

(d)

(b)

(a)

**Figure S2** Seasonal (JJA) climate change effects in the reference simulation when climate changes from (a, c, e) IPSL-CM5A-MR and (b, d, f) GFDL-ESM2M are applied. (a, b) relative precipitation changes ($\Delta_{r}^{CC}TP$) [%]; (c, d) relative soil moisture fraction changes ($\Delta_{r}^{CC}X$) [%]; (e, f) absolute changes in the temporal correlation coefficient (${\Delta^{CC}r}_{X}^{ET}$) [-] between daily evapotranspiration ($ET$) and $X$ from May to September. Distribution, median, and percentile range (“(10-90)%”) across the study area (b/w dashed polygon) are given to the right of each subfigure.


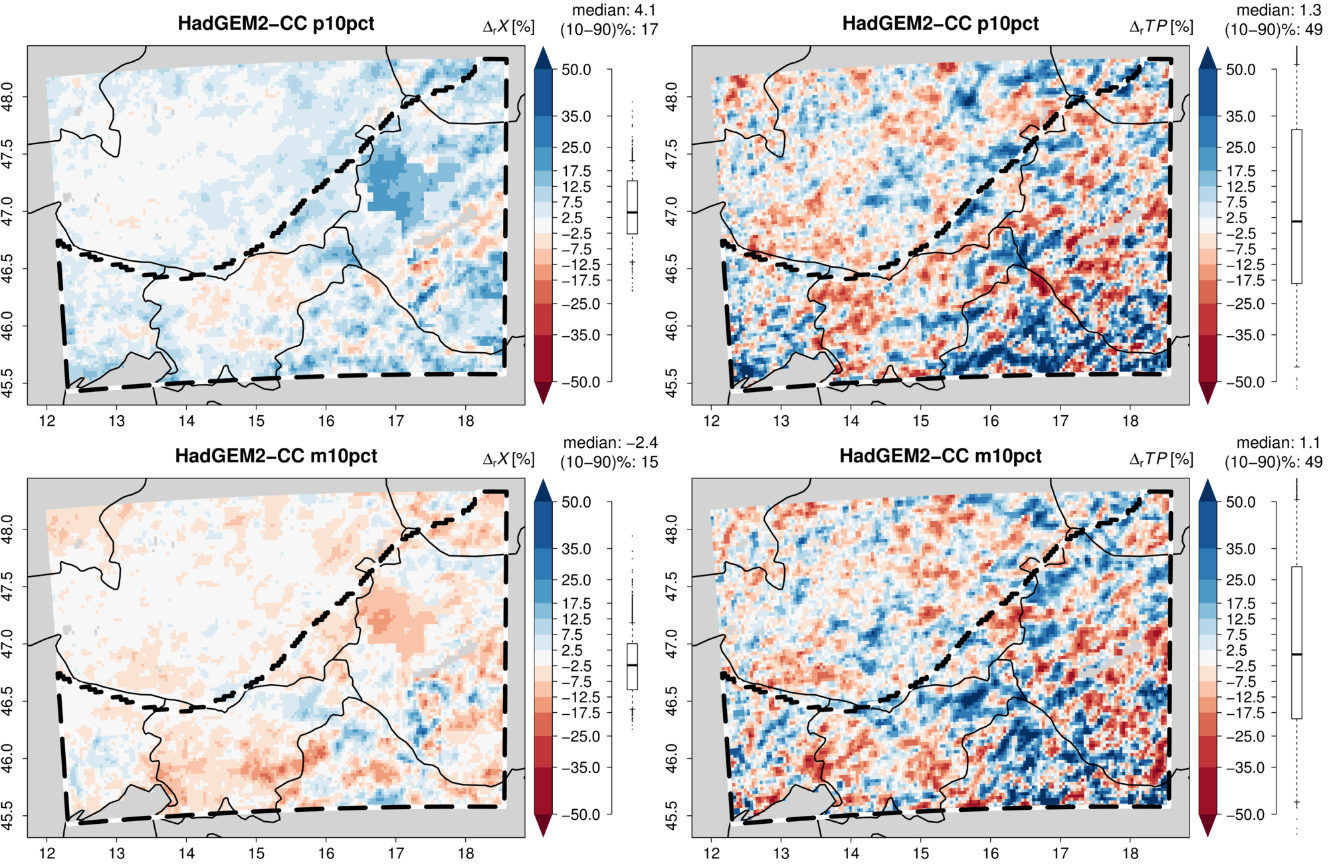


(d)

(c)

(b)

(a)

**Figure S3** Relative JJA deviations ($\Delta_{r}$) [%] of the (a, b) p10pct and (c, d) m10pct simulations from the reference simulation with climate changes from HadGEM2-CC. (a, c) deviations in soil moisture fraction ($X$); (b, d) total precipitation ($TP$). Distribution (boxplot), median, and percentile range (“(10-90)%”) across the study area (b/w dashed polygon) are given to the right of each subfigure.


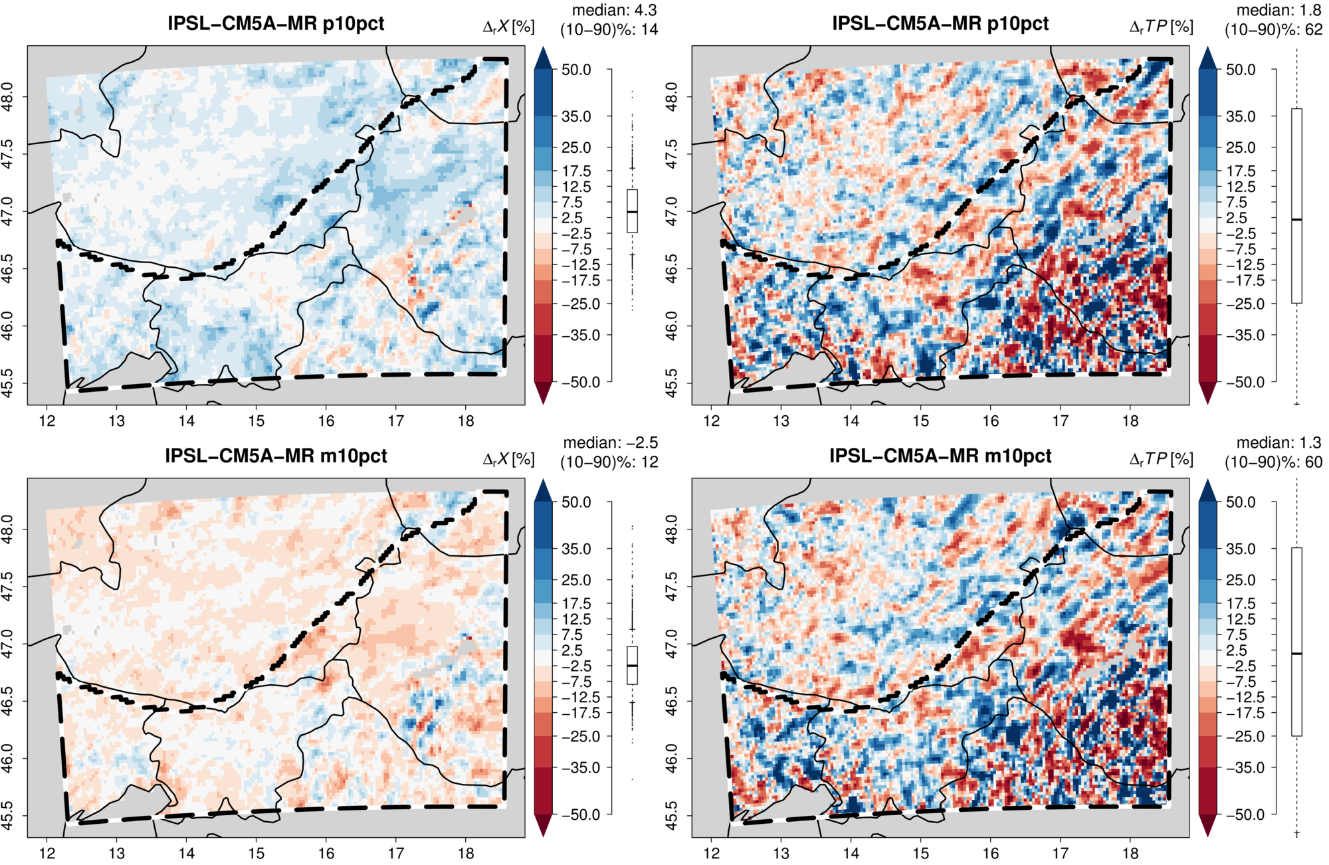


(d)

(c)

(b)

(a)

**Figure S4** Same as Figure S3, but for IPSL-CM5A-MR.

(d)

(c)

(b)

(a)


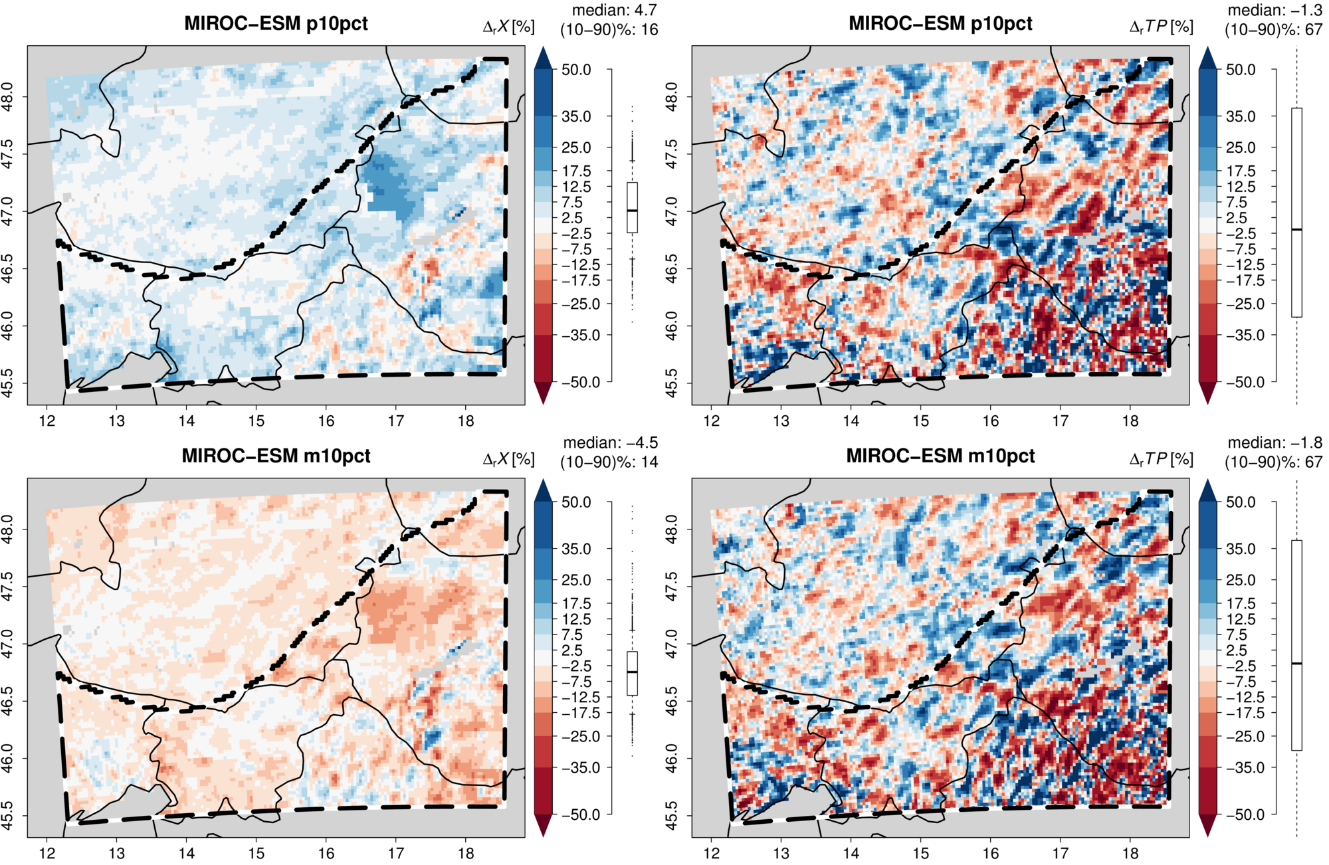


**Figure S5** Same as Figure S3, but for MIROC-ESM.


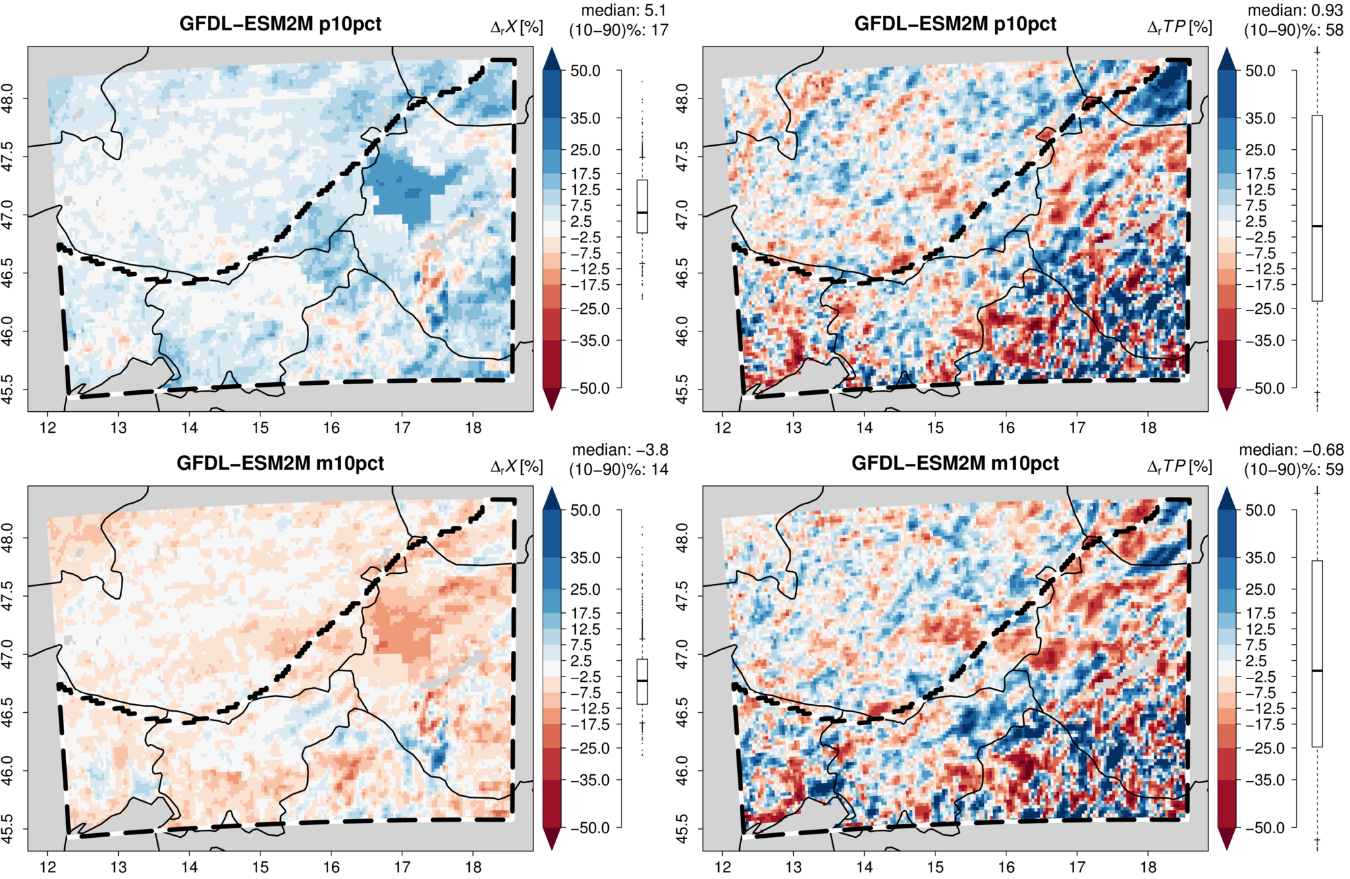


(d)

(b)

(c)

(a)

**Figure S6** Same as Figure S3, but for GFDL-ESM2M.


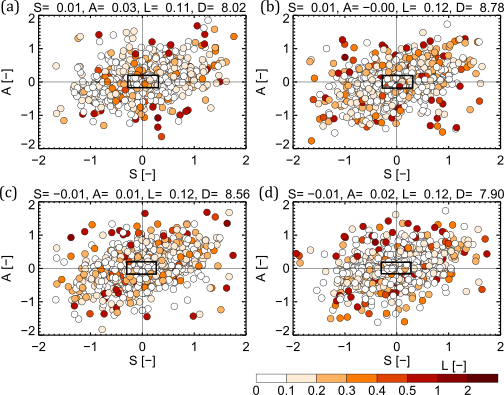


**Figure S7** Scatter plot of the structure ($S$) and amplitude ($A$) components for JJA from comparing each precipitation field (1210 in total) of (a) p10pct, (b) p5pct, (c) m5pct, (d) m10pct with the reference simulation under future climate conditions from HadGEM2-CC. The location ($L$) component is given by the color. The rectangle in the center indicates the interquartile range (50 % of all data) of $S$ and $A$. Mean values of $S$, $A$, and $L$ are given above each subfigure. $D$ gives the average minimum distance (in multiples of the grid spacing) between corresponding precipitation objects. The standard deviation across the perturbed simulations ($\sigma$) of $D$ gives 0.42.


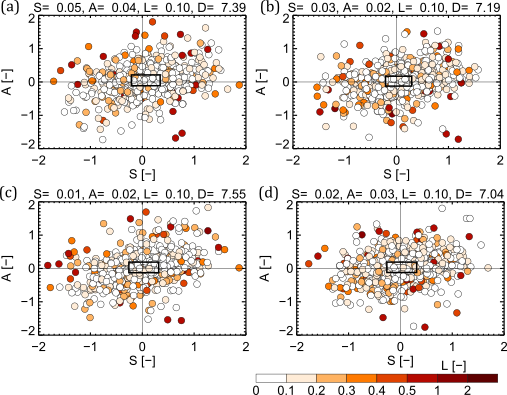


**Figure S8** Same as Figure S7 but for IPSL-CM5A-MR, with 960 events and $\sigma=0.22$.


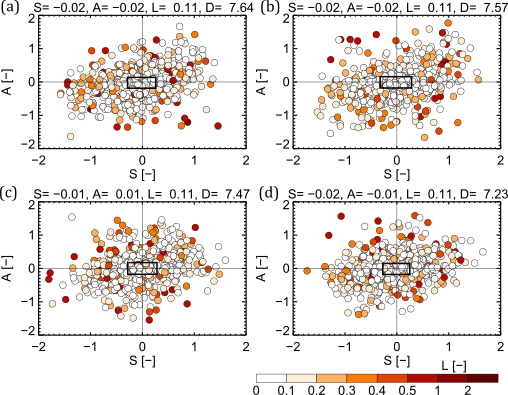


**Figure S9** Same as Figure S7 but for MIROC-ESM, with 910 events and $\sigma=0.18$.


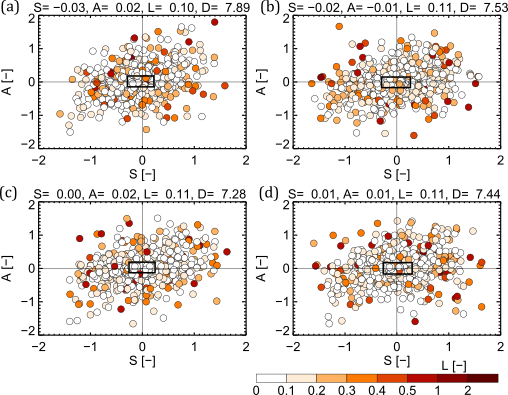


**Figure S10** Same as Figure S7 but for GFDL-ESM2M, with 990 events and $\sigma=0.26$.


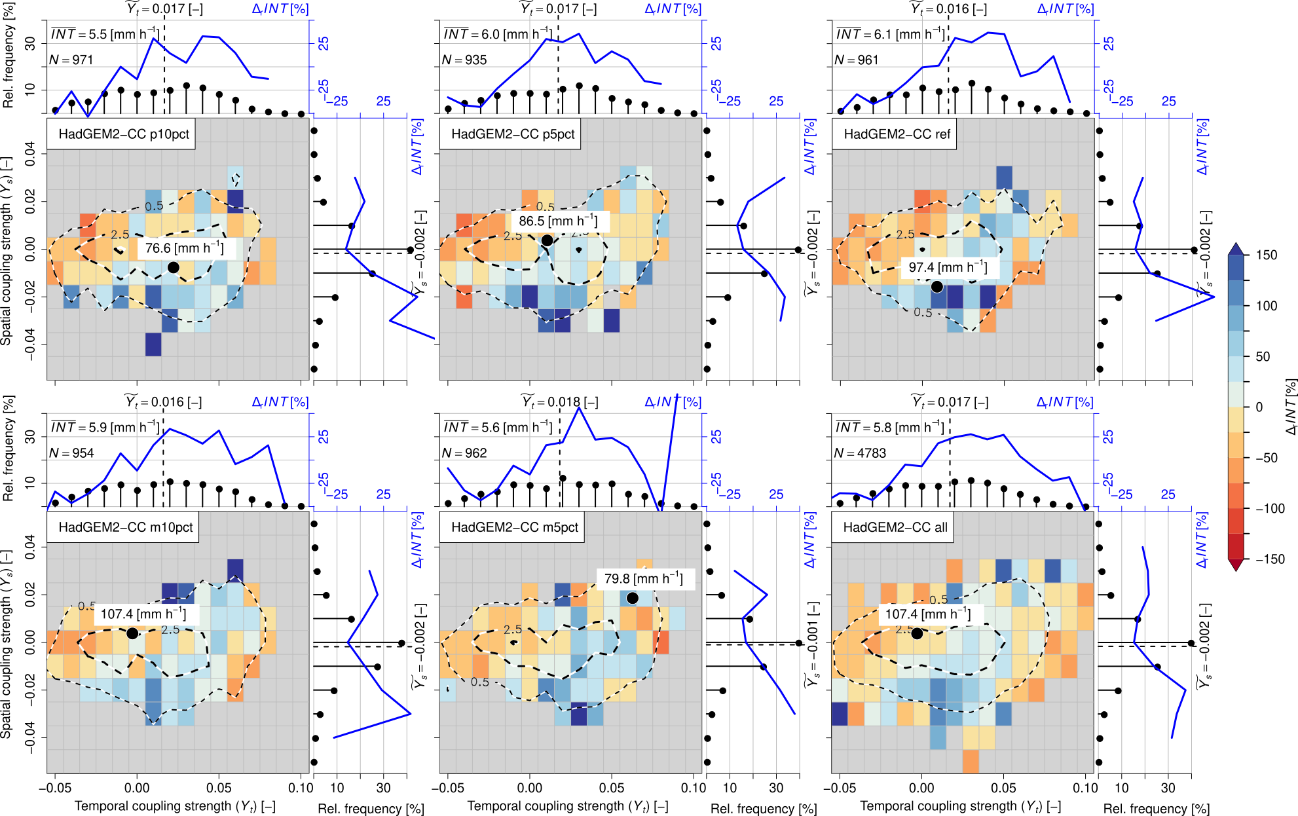


(c)

(f)

(b)

(a)

(d)

(e)

**Figure S11** Interplay between spatial and temporal SMP coupling strengths ($Y_{s}$ and $Y_{t}$, respectively) and maximum hourly precipitation intensities ($INT$) of $N$ isolated precipitation events during JJA in the study area with thermodynamic climate changes from HadGEM2-CC. The color shaded area depicts estimated intensity anomalies ($\Delta_{r}INT$) from bins (with a width of 0.01) of $Y_{s}$ and $Y_{t}$ related to the given averaged maximum intensity ($\bar{INT}$). The maximum $INT$ of all events (black dot) is given. The dashed contour lines at levels of 0.5 % and 2.5 % of the underlying bivariate percent relative frequency distribution of $Y_{s}$ and $Y_{t}$ are shown. Marginal percent relative frequency distributions of $Y_{s}$ and $Y_{t}$ (black dots) together with corresponding percent relative intensity anomalies (blue line) are shown at the top and to the right of each subfigure. Median feedback strengths ($\tilde{Y_{s}}$, $\tilde{Y_{t}}$) are given. (a) simulation p10pct, (b) p5pct, (c) ref, (d) m10pct, (e) m5pct, (f) all simulations pooled together (referred to as “all”).


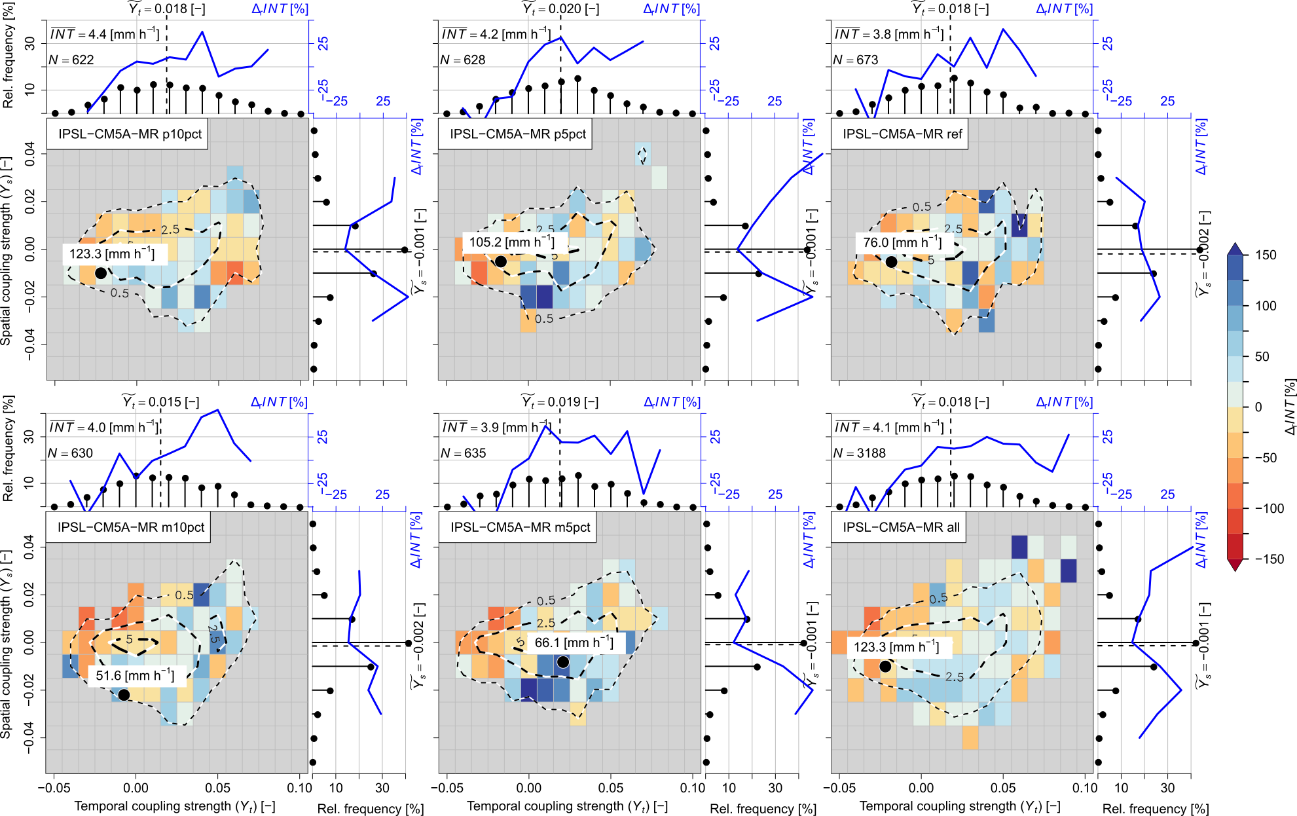


(c)

(f)

(b)

(a)

(d)

(e)

**Figure S12** Same as Figure S11, but for IPSL-CM5A-MR.


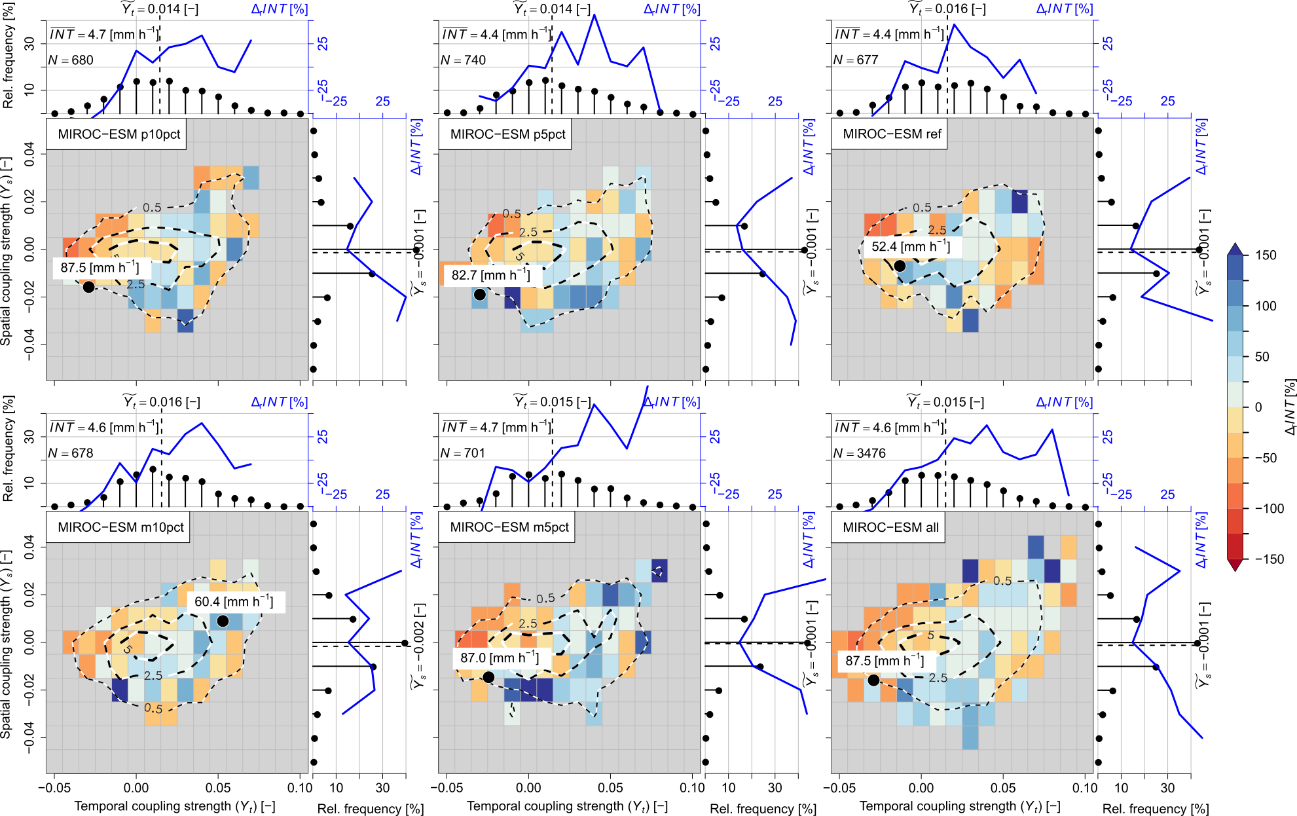


(c)

(f)

(b)

(a)

(d)

(e)

**Figure S13** Same as Figure S11, but for MIROC-ESM.


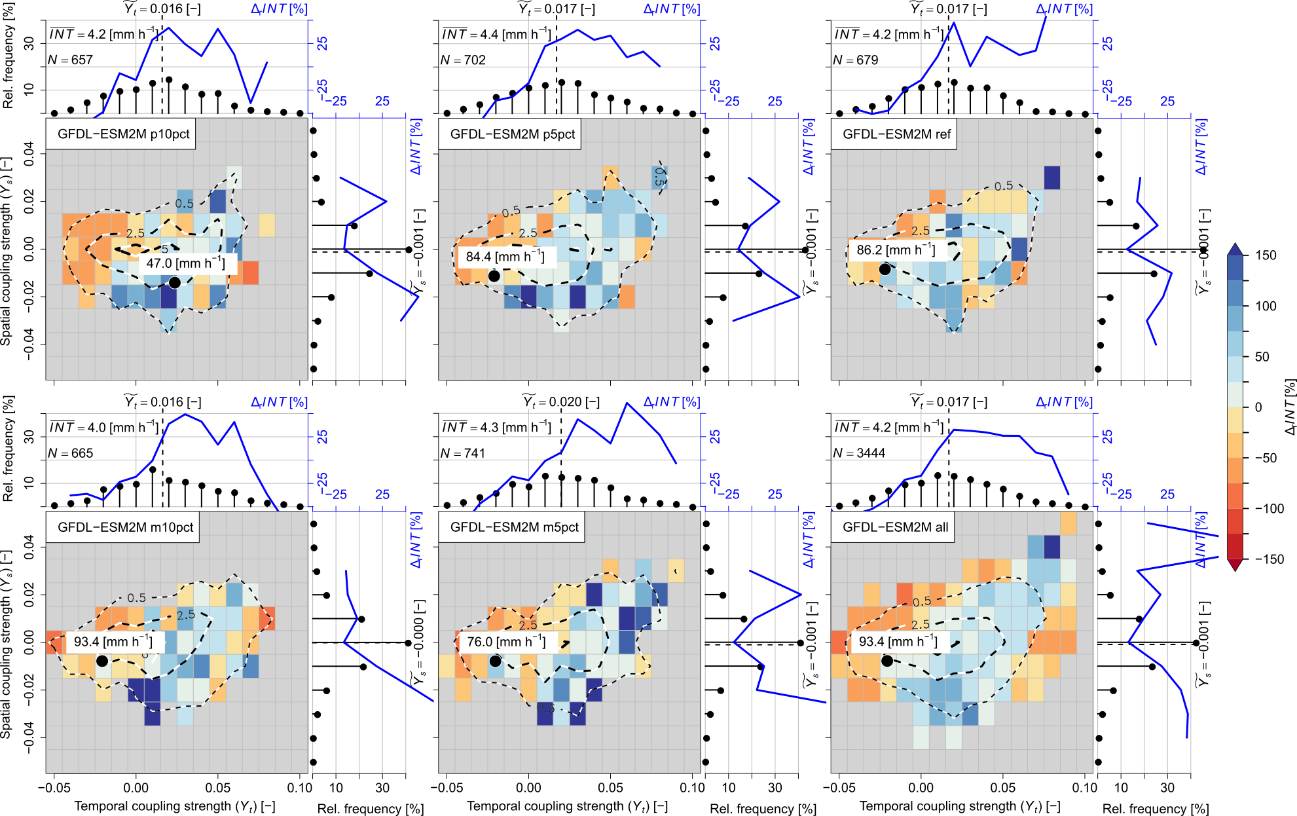


(c)

(f)

(b)

(a)

(d)

(e)

**Figure S14** Same as Figure S11, but for GFDL-ESM2M.

# Tables

**Table S1** Table of conducted simulations. Matrix of conducted (marked with “x”) combinations of percent relative deep soil moisture perturbations and driving data, including the GCMs used for pseudo global warming.

| Perturbation Driving data | 0 % ref | +10 % p10pct | +5 % p5pct | -5 % m5pct | -10 % m10pct |
| --- | --- | --- | --- | --- | --- |
| IFS | x | x | x | x | x |
| HadGEM2-CC | x | x | x | x | x |
| IPSL-CM5A-MR | x | x | x | x | x |
| MIROC-ESM | x | x | x | x | x |
| GFDL-ESM2M | x | x | x | x | x |

**Table S2** Study area median ($\tilde{∎}$) and percentile range ($∎_{10}^{90}$) of seasonal (JJA) $X$ [-] and $TP$ [mm d^-1^] of the reference and the perturbation simulations under current climate conditions (driving data IFS).

| Simulation acronym | $\tilde{X}$[-] | ${(X)}_{10}^{90}$[-] | $\tilde{TP}$[mm d^-1^] | ${(TP)}_{10}^{90}$[mm d^-1^] |
| --- | --- | --- | --- | --- |
| ref | 0.17 | 0.29 | 3.2 | 3.1 |
| p10pct | 0.18 | 0.30 | 3.2 | 3.2 |
| p5pct | 0.18 | 0.29 | 3.2 | 3.1 |
| m5pct | 0.17 | 0.28 | 3.2 | 3.1 |
| m10pct | 0.16 | 0.28 | 3.2 | 3.1 |
